# A six-metabolite panel as potential blood-based biomarkers for Parkinson’s disease

**DOI:** 10.1038/s41531-021-00239-x

**Published:** 2021-10-14

**Authors:** Stephan Klatt, James D. Doecke, Anne Roberts, Berin A. Boughton, Colin L. Masters, Malcolm Horne, Blaine R. Roberts

**Affiliations:** 1 The Florey Institute of Neuroscience and Mental Health, The University of Melbourne, Parkville, VIC 3052 Australia; 2 Cooperative Research Centre for Mental Health, Parkville, VIC 3052 Australia; 3 Australian e-Health Research Centre, CSIRO, Brisbane, QLD Australia; 4 Department of Biochemistry, Emory University School of Medicine, Atlanta, GA 30322 USA; 5 School of Biosciences, The University of Melbourne, Parkville, VIC 3052 Australia; 6 Australian National Phenome Centre, Murdoch University, Murdoch, WA 6150 Australia; 7 Department of Neurology, Emory University School of Medicine, Atlanta, GA 30322 USA

**Keywords:** Diagnostic markers, Metabolomics, Parkinson's disease

## Abstract

Characterisation and diagnosis of idiopathic Parkinson’s disease (iPD) is a current challenge that hampers both clinical assessment and clinical trial development with the potential inclusion of non-PD cases. Here, we used a targeted mass spectrometry approach to quantify 38 metabolites extracted from the serum of 231 individuals. This cohort is currently one of the largest metabolomic studies including iPD patients, drug-naïve iPD, healthy controls and patients with Alzheimer’s disease as a disease-specific control group. We identified six metabolites (3-hydroxykynurenine, aspartate, beta-alanine, homoserine, ornithine (Orn) and tyrosine) that are significantly altered between iPD patients and control participants. A multivariate model to predict iPD from controls had an area under the curve (AUC) of 0.905, with an accuracy of 86.2%. This panel of metabolites may serve as a potential prognostic or diagnostic assay for clinical trial prescreening, or for aiding in diagnosing pathological disease in the clinic.

## Introduction

Parkinson’s disease (PD) is the second most common neurodegenerative disease after Alzheimer’s disease (AD) and affects around five million people worldwide^1^. Neuropathological hallmarks of PD include; the loss of catecholaminergic neurons in the substantia nigra, an increase in striatal dopamine deficiency and the presence of ɑ-synuclein aggregate-containing intracellular inclusions^2^. Moreover, the abundance, structure and function of the striatal *N*-Methyl-d-Aspartate (NMDA) receptor is altered by the dopamine depletion and pharmacological treatments used in PD^3^. NMDA receptors are ion channel proteins composed of multiple subunits and allow positively charged ions (like Zn^2+^, Mg^2+^ and Ca^2+^) to pass the cell membrane when activated via glutamate and glycine binding. They have complex regulatory properties and play a central role in synaptic plasticity, learning and memory^4^. Another potential player in the pathogenesis of PD is the RhoA-ROCK pathway. It plays a critical role in inflammation, and e.g. ROCK inhibitors may provide new protective strategies against PD progression^5,6^. As is common for diseases where the aetiology is unknown, PD is diagnosed clinically with the presence of^7^ bradykinesia, supported by the presence of rest tremor, postural instability and rigidity^8,9^. Non-motor symptoms such as disruption of gastric tract motility (constipation), sleep disturbance and depression are frequently present. The accuracy of clinical diagnosis has been well described^10,11^ and the clinical phenotype, especially at the onset of the disease, can encompass more than one pathophysiological entity. Since its first description by James Parkinson in 1817, the disease has been split into several different entities, including idiopathic PD (iPD). However, the severity and progression of iPD varies and it is uncertain whether this variation indicates further sub-entities or a broad range of phenotypes of a single entity.

People with PD usually present with symptoms when 50% or more dopaminergic neurons of the substantia nigra are lost^12–14^. Disease-modifying therapies would be therefore most effective when introduced early. This would be ideally prior to significant neuronal loss and thus well before clinical manifestations were apparent. While there are currently no disease-modifying therapies for PD, early and accurate identification of PD would aid in the discovery of such therapies. Furthermore, research into the understanding of the early pathophysiological event in PD would be aided by presymptomatic recognition.

Currently, no reliable biomarker exist that detect presymptomatic iPD. This underlines the importance of the development of a new diagnostic marker to facilitate both early diagnosis and assessment of new potential treatments. One key phenotype associated with iPD is a pronounced presence of oxidative stress markers including nitration and oxidation^15,16^. Altered levels of metabolites, including those associated with oxidation, have been measured in a variety of sample types (i.e. brain tissue^17,18^, cerebrospinal fluid (CSF)^19–21^, blood serum^22,23^, blood plasma^24,25^, red blood cells (RBC)^26,27^, sebum^28^ and urine^29^) from both drug-naïve and l-3,4-dihydroxyphenylalanine (l-DOPA) treated iPD patients with samples taken from healthy, age-matched control groups. Affected metabolic pathways include the tryptophan/kynurenine catabolic pathway (KP)^17,19,24,30–32^, polyamine pathway^27,33,34^, glutathione synthesis pathway^35–37^, lipids and lipid (per)oxidation^23,38–42^, fatty acid- and beta oxidation^43–45^, purine pathway^22,46–48^, energy metabolism^20,49^ as well as concentration changes of most proteinogenic amino acids^25,29,50–53^. Metabolites of the kynurenine and polyamine pathways have been found to be neuroprotective^54–57^ or neurotoxic^19,58–60^, and small changes to them can substantially disturb pathway equilibrium^61^. Moreover, both pathways are interconnected as some of their key players have been shown to bind and alter glutaminergic signalling of the NMDA receptor^62,63^.

In this study, we used a targeted triple quadrupole liquid chromatography-mass spectrometry (QQQ LC/MS) approach to quantify the concentration of 38 iPD-relevant metabolites extracted from the blood serum of 231 individuals to uncover changes solely based on the disease. Included metabolites are 20 proteinogenic amino acids, several metabolites of the kynurenine pathway (KP) including l-Kynurenine (l-KYN), 3-hydroxy-l-kynurenine (3-HK), 3-hydroxyanthranillic acid (3-AA) and the polyamines (PAs) cadaverine-2 (Cad) and putrescine-2 (Put). Metabolites that contain one or two amine groups were derivatized with 6-aminoquinolyl-*N*-hydroxysuccinimidyl carbamate (AQC). This reagent is used to increase detection of the amine in the mass spectrometer and enables standard reverse-phase chromatography^64^. We analyzed the influence of l-DOPA on the tested metabolites as well as the influence of age and sex prior and after confounder adjustment. In addition to this, we investigated changes in the ratios and interactions of all targeted metabolites and used this to identify potential biomarkers. The analysed cohort is one of the largest so far (e.g. see supplement from Stoessel et al. (2018)), containing 103 l-DOPA treated iPD patients, 7 drug-naïve iPD patients (dn-iPD), 93 healthy age-matched controls and 28 patients with AD as a disease-specific control group. Using this cohort, we hypothesised that metabolites could serve as the basis of a diagnostic assay.

## Results

### Amino acid and metabolite differences between CN, iPD and AD groups

**Fig. 1:**
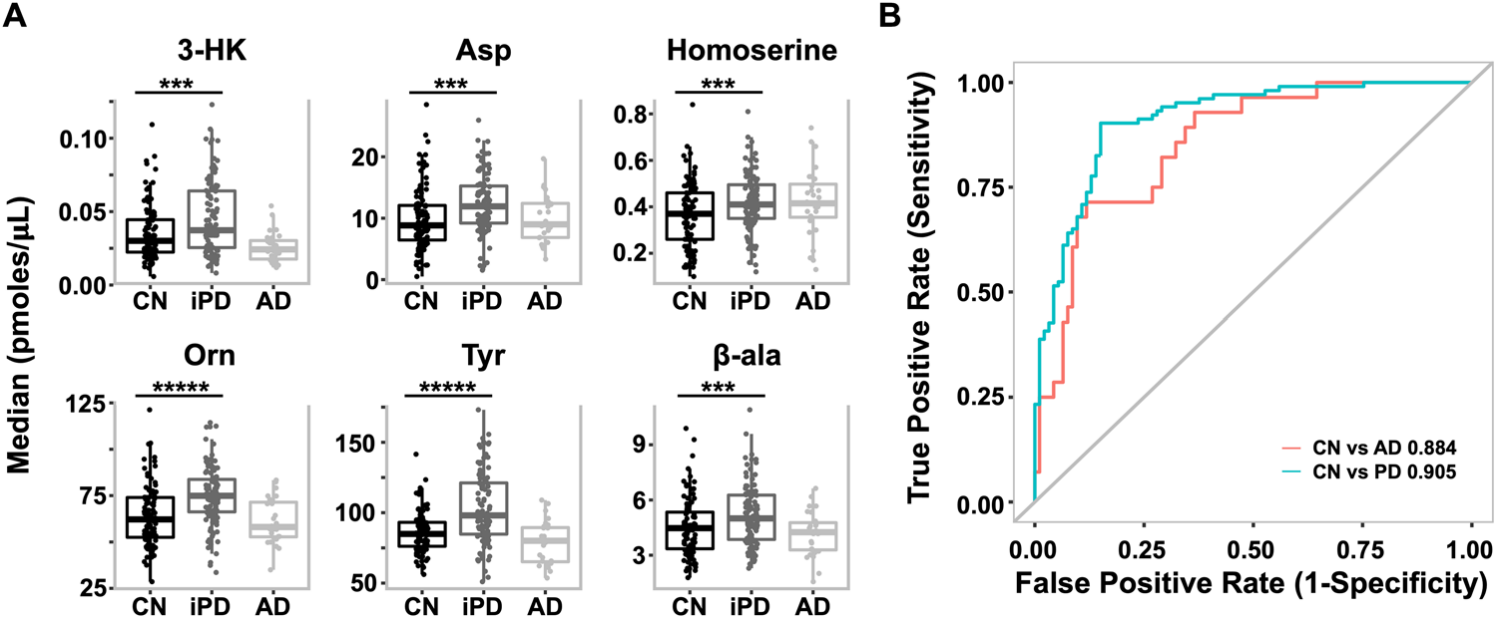
Elevation of six metabolites in iPD serum. **A** Box plot of top six biomarker iPD vs CN and AD (median values in picomoles/µl serum, data taken from Table 1). *** indicates a *p* value of <0.001 and ***** of <0.00001. **B** Multivariate ROC analyses resulted in a linear model being able to separate iPD from CN and AD from CN. In the case of iPD vs CN, a panel of seven metabolites resulted in an AUC value of 0.905. For further details, see Table 2C.

The mean metabolite concentration (in picomoles/µL of serum) and standard deviation (SD) for each metabolite by the group is summarised in Table 1. For the control (CN) vs iPD comparisons, there were 11 significant concentration differences (*p* < 0.001) prior to adjustment for age and sex. After adjustment, only six remained statistically significant; with 3-HK, l-aspartic acid (Asp), β-alanine (β-ala), homoserine, ornithine (Orn) and tyrosine (Tyr) all being increased in the iPD group as compared with the CN group (Fig. 1). Moreover, there were five metabolites (3-HK, l-cysteine (Cys), l-glutamic acid (Glu), Orn and Tyr) that showed significant differences between the AD and iPD groups after adjustment for both confounders and multiple comparisons, with Cys as the only significantly increased metabolite in AD (Supplementary Table 1). Comparison between CN and AD revealed no significant differences. This is most likely due to the limited power in the AD cohort (*n* = 28).

**Table 1:** Mean metabolite concentrations (pmol µL^−1^) with standard deviations (SD) and results of pairwise comparison prior and after the adjustment for age and sex.

Metabolite	Mean (SD); in picomoles/μL				Unadjusted for confounders *p* values		Adjusted for confounders *p* values	
	CN (*n* = 93)	naïve-iPD (*n* = 7)	iPD (*n* = 103)	AD (*n* = 28)	iPD vs CN	AD vs CN	iPD vs CN	AD vs CN
2-Ambut	22.03 (6.72)	24.34 (9.77)	20.7 (5.33)	20.49 (8.59)	0.42	0.21	0.15	0.15
3-AA	0.04 (0.01)	0.03 (0.01)	0.04 (0.02)	0.03 (0.01)	0.04	0.004	0.19	0.01
3-HK	0.04 (0.02)	0.05 (0.06)	0.05 (0.04)	0.03 (0.01)	**4.00E-05**	0.002	**3.96E-04**	0.01
4-OH-Pro	11.37 (5.01)	13.97 (6.83)	12.16 (4.94)	12.41 (5.35)	0.19	0.38	0.23	0.15
Ala	414.07 (81.01)	438.91 (119.44)	432.91 (90.87)	407.2 (80.2)	0.16	0.69	0.16	0.71
Arg	81.5 (20.04)	76.93 (24.66)	73.81 (15.54)	85.11 (19.45)	0.04	0.36	0.34	0.50
Asn	41.65 (6.23)	42.59 (7.95)	42.59 (6.61)	38.65 (4.35)	0.20	0.01	0.49	0.14
Asp	9.69 (5.32)	10.81 (5.71)	12.57 (4.99)	9.83 (3.82)	**5.07E-04**	0.63	**1.13E-03**	0.87
β-ala	4.52 (1.59)	4.69 (1.96)	5.19 (1.64)	4.41 (1.8)	**1.16E-03**	0.67	**1.20E-03**	0.81
Cad	0.2 (0.09)	0.13 (0.07)	0.17 (0.08)	0.22 (0.1)	**5.92E-04**	0.45	0.02	0.74
Citrulline	34.61 (8.12)	32.03 (7.7)	33.06 (7.22)	37.06 (9.57)	0.16	0.37	0.76	0.55
Cys	177.14 (28.59)	160.55 (29.36)	157.78 (27.56)	191.13 (22.99)	**4.00E-06**	0.01	0.06	0.04
GABA	0.32 (0.17)	0.34 (0.19)	0.31 (0.17)	0.35 (0.16)	0.50	0.27	0.73	0.53
Gln	710.1 (81.81)	729.43 (140.56)	690.75 (80.77)	711.35 (65.59)	0.15	0.84	0.20	0.42
Glu	45.94 (20.46)	52.99 (13.61)	54.88 (20.47)	38.74 (15.61)	**3.05E-04**	0.09	0.002	0.12
Gly	274.57 (67.96)	268.57 (63.31)	298.52 (86.63)	294.61 (61.72)	0.05	0.04	0.01	0.07
His	70.47 (12.75)	75.28 (8.27)	70.58 (11.31)	69.25 (11.27)	0.98	0.60	0.55	0.80
Homoserine	0.37 (0.14)	0.42 (0.11)	0.43 (0.13)	0.42 (0.15)	**3.48E-04**	0.11	**1.39E-03**	0.11
Ile	78.89 (14.87)	81.76 (11.6)	83.54 (17.3)	74.58 (19.6)	0.12	0.10	0.60	0.38
l-KYN	3.02 (0.78)	2.45 (0.7)	2.59 (0.7)	2.69 (0.94)	**4.70E-05**	0.04	0.02	0.01
Leu	156.23 (28.16)	167.94 (29.47)	165.33 (34.58)	139.5 (25.99)	0.12	0.005	0.75	0.06
Lys	229.23 (33.62)	219.13 (10.11)	222.4 (36.23)	214.97 (35.57)	0.23	0.06	0.30	0.09
Met	29.94 (4.36)	30.21 (3.13)	30.62 (5.23)	27.73 (4.29)	0.33	0.02	0.92	0.13
*N*-acetyl-phenylalanine	0.03 (0.01)	0.02 (0)	0.03 (0.01)	0.02 (0.01)	0.01	0.01	0.11	0.06
Orn	63.82 (16.44)	66.93 (26.17)	74.95 (16.14)	61.41 (12.1)	**3.00E-06**	0.58	**1.40E-05**	0.77
Phe	79.92 (9.29)	80.7 (10.2)	83.44 (11.08)	75.63 (8.83)	0.01	0.03	0.14	0.16
Pro	228.05 (54.83)	274.65 (98.72)	246.8 (76.35)	259.25 (83.25)	0.08	0.09	0.26	0.01
Put	0.28 (0.15)	0.23 (0.07)	0.24 (0.18)	0.29 (0.15)	0.02	0.66	0.05	0.78
Sarco	1.8 (0.63)	2.08 (0.89)	1.83 (0.61)	1.7 (0.66)	0.98	0.25	0.17	0.64
Ser	89.3 (17.26)	86.46 (16.05)	92.55 (18.43)	91.36 (16.96)	0.14	0.53	0.15	0.44
Serotonin	0.39 (0.22)	0.34 (0.21)	0.43 (0.32)	0.33 (0.24)	0.75	0.09	0.69	0.07
Tau	111.79 (31.34)	124.97 (35.26)	124.91 (32.11)	121.06 (28.68)	**1.22E-03**	0.20	0.01	0.37
Thr	125.32 (23.7)	122.05 (20.64)	137.59 (31.11)	122.77 (26.25)	0.003	0.58	0.01	0.96
Trp	75.04 (13.19)	78.18 (10.4)	74.46 (12.72)	65.49 (12.27)	0.89	**1.18E-03**	0.14	0.01
Tryptamine	0.02 (0.01)	0.02 (0.01)	0.02 (0.01)	0.02 (0.01)	0.58	0.02	0.95	0.03
Tyr	86.02 (14.66)	88.82 (7.86)	102.73 (25.51)	78.74 (15.1)	**<1.00E-4**	0.03	**9.00E-06**	0.05
Val	276.45 (47.38)	292.45 (31.63)	283.03 (53.43)	248.16 (46.45)	0.33	0.01	0.78	0.03

Significant *p* values of less than 0.001 are indicated in bold.

### The influence of age and sex on metabolite concentrations

Correlations between metabolites and with age are shown in Supplementary Table 2. Weak to moderate significant correlations were found between age and l-arginine (Arg; *R* = 0.25, *p* < 0.001), Cad (*R* = 0.22, *p* < 0.001), Citrulline (*R* = 0.32, *p* < 0.001), Cys (*R* = 0.50, *p* < 0.001), l-leucine (Leu; *R* = −0.26, *p* < 0.001) and l-KYN (*R* = 0.31, *p* < 0.001). Comparing metabolite concentrations between males and females found that l-asparagine (Asn), β-ala, 4-hydroxyproline (4-OH-Pro), l-isoleucine (Ile), Leu, l-methionine (Met), l-proline (Pro), sarcosine (Sarco), l-tryptophan (Trp) and l-valine (Val) had significantly higher levels in males as compared to females (*p* < 0.001), while only 3-AA was found to have significantly higher levels in females. There were, however, no significant interactions between either age and CN/iPD or between gender and CN/iPD associated with metabolites (assessed via generalised linear modelling (GLM)).

### ROC curve analyses, iPD biomarker performance evaluation and diagnostic tests

We performed receiver operating characteristic (ROC) analyses to determine the AUC (area under the curve) of the top-ranked serum metabolites to predict either AD or iPD participants from healthy controls. For the CN vs iPD comparison, 16 individual metabolites had AUC values ranging between 0.591 and 0.706 with a *p* value of <0.05 (Table 2A). For the CN group vs AD comparisons, 11 individual metabolites had AUC values ranging between 0.629 and 0.706 with a *p* value <0.05 (Table 2B).

**Table 2:** ROC curve analysis for biomarker performance evaluation and diagnostic test results, including ROC curve with a 95% confidence interval, *p* value, sensitivity, specificity, positive predicted value (PPV), negative predicted value (NPV) and accuracy (ACC).

(A) iPD vs CN (top 16)	AUC 95%CI	*p* value	Sensitivity	Specificity	PPV	NPV	Accuracy
Cys	0.705 (0.63–0.78)	5.04E-07	64.55	68.82	71	62.14	66.5
Tyr	0.698 (0.63–0.77)	1.16E-06	55.45	75.27	72.62	58.82	64.53
Orn	0.689 (0.62–0.76)	3.40E-06	72.73	62.37	69.57	65.91	67.98
KYN	0.664 (0.59–0.74)	5.67E-05	68.18	62.37	68.18	62.37	65.52
Asp	0.662 (0.59–0.74)	7.08E-05	80.91	50.54	65.93	69.12	67
3-OH KYN	0.641 (0.56–0.72)	5.62E-04	40	89.25	81.48	55.7	62.56
Cad	0.639 (0.56–0.72)	6.29E-04	60	61.29	64.71	56.44	60.59
Glu	0.638 (0.56–0.71)	6.97E-04	64.55	58.06	64.55	58.06	61.58
Tau	0.635 (0.56–0.71)	9.40E-04	76.36	52.69	65.63	65.33	65.52
Homoserine	0.635 (0.56–0.71)	9.35E-04	83.64	38.71	61.74	66.67	63.05
beta-Ala	0.62 (0.54–0.7)	3.27E-03	52.73	66.67	65.17	54.39	59.11
Thr	0.603 (0.53–0.68)	1.12E-02	70	49.46	62.1	58.23	60.59
*N***-**Acetyl-phenylalanine	0.601 (0.52–0.68)	1.31E-02	68.18	53.76	63.56	58.82	61.58
Phe	0.597 (0.52–0.68)	1.69E-02	43.64	74.19	66.67	52.67	57.64
Put	0.591 (0.51–0.67)	2.58E-02	40	81.72	72.13	53.52	59.11
Arg	0.583 (0.5–0.66)	4.15E-02	52.73	61.29	61.7	52.29	56.65
(B) AD vs CN (top 11)							
Trp	0.706 (0.6–0.82)	9.76E-04	46.43	88.17	54.17	84.54	78.51
3-OH ANA	0.686 (0.58–0.79)	2.99E-03	75	56.99	34.43	88.33	61.16
Val	0.672 (0.55–0.79)	6.07E-03	78.57	56.99	35.48	89.83	61.98
Leu	0.662 (0.54–0.78)	9.76E-03	64.29	64.52	35.29	85.71	64.46
3-OH KYN	0.66 (0.55–0.77)	1.07E-02	92.86	38.71	31.33	94.74	51.24
KYN	0.652 (0.52–0.78)	1.51E-02	67.86	61.29	34.55	86.36	62.81
Cys	0.649 (0.54–0.76)	1.70E-02	89.29	39.78	30.86	92.5	51.24
Phe	0.647 (0.53–0.76)	1.90E-02	78.57	55.91	34.92	89.66	61.16
Typtamine	0.646 (0.53–0.77)	1.97E-02	75	59.14	35.59	88.71	62.81
Asn	0.644 (0.54–0.75)	2.10E-02	82.14	53.76	34.85	90.91	60.33
Tyr	0.629 (0.5–0.76)	3.92E-02	39.29	88.17	50	82.83	76.86
(C) Seven-marker model							
Cys, 2-Ambut, Tyr, KYN, Arg/3-AA, Asp/KYN, beta-Ala*Orn	0.905	<0.0001	87.4	85.0	86.5	85.9	86.2

Results for the binary classification of (A) iPD vs CN (top 16 metabolites), (B) AD vs CN (top 11 metabolites) and (C) the seven-marker model for iPD vs CN is shown.

### Ratios and interactions that separate CN from iPD and AD disease groups

In neurodegenerative diseases, the ratio of markers associated with the pathology (e.g. tau, amyloid beta) often serve as more powerful biomarkers than the absolute level of the biomarker alone. One clear example of this is the ratio of cerebral spinal fluid amyloid beta 1–42 peptide to tau protein levels^65,66^. We hypothesised that this would also be the case for metabolites. To test this, all possible ratios and interactions (the product of two analytes) for the 37 metabolites (excluding l-DOPA) and amino acids were computed providing 1332 markers. Dimension reduction via removal of those ratios/interactions with low variance (SD <0.5) reduced this number to 569. Comparing the mean ratio/interaction levels between the CN and iPD groups, we identified 11 ratios and 23 interactions that were significantly altered (*p* < 0.00009) between the two groups (Supplementary Table 3). Of these, Asp was involved with the most ratios (7/11) and interactions (9/23). Other metabolites that appeared frequently in the top 34 included l-glutamine (Gln; five interactions), homoserine (four interactions), 3-HK (four interactions and one ratio) and Orn (six interactions and one ratio). Of these 34 markers, seven remained significant post adjusting for age and gender (homoserine*Orn, β-ala*Orn, Asp*Tyr, Gln*Tyr, Asp*Orn, Asp / L-KYN and Gln*Orn).

### Multivariate analyses of metabolites for iPD and AD

Following up the univariate assessment of metabolites, we conducted a multivariate analysis including both individual analytes and ratio’s/interactions to see if a panel of markers together could provide better discrimination between CN and disease groups. Using a combination of feature selection (LASSO) and model selection via Akaike information criterion (AIC) reduction, seven markers were selected in a linear model to separate CN from iPD participants (Cys [*p* = 0.008], 2-aminobutyric acid (2-Ambut) [*p* = 0.0002], Tyr [*p* = 0.0005], l-KYN [*p* = 0.0003], ratio of Arg/3-AA [*p* = 0.004], ratio of Asp/l-KYN [*p* = 0.007] and product of β-ala*Orn [*p* < 0.0001]). These seven metabolites resulted in an AUC value of 0.905 with an accuracy of 86.2% (sensitivity: 87.4%, specificity: 85.0%, positive predicted value (PPV): 86.5% and negative predicted value (NPV): 85.9%, Table 2C and Fig. 1B). For the comparison between CN and AD participants, using the same method to define a multivariate set of analytes, we identified a set of six markers (Asp [*p* = 0.019], Cys [*p* = 0.0008], Tryp [*p* = 0.022], Homoserine/*N*-Acetyl-phenylalanine [*p* = 0.055], Pro/3-HK [*p* = 0.002] and Gln*Typtamine [*p* = 0.063]) that worked together to separate AD from CN participants. Here, ROC analyses calculated an AUC of 0.884 to predict AD from CN with 79.3% accuracy (sensitivity: 89.3%, specificity: 76.3%, PPV: 53.2% and NPV: 95.9%).

## Discussion

While proteins are recognised as playing important roles in iPD and its progression^67^, there is increasing recognition of the importance of metabolites in the disease phenotype^68^. In the current study, we quantified 37 iPD-relevant metabolites (- l-DOPA) from the blood serum of 231 individuals with the goal to find potential biomarkers to separate CN from the disease. l-DOPA was removed from the statistics, as it results in the most prominent and expected change. After adjustment for age and sex, a multivariate analysis followed by ROC predictions defined a biomarker panel of four metabolites (Cys, 2-Ambut, Tyr, l-KYN), two ratios (Arg/3-AA, Asp/l-KYN) and one interaction (β-ala*Orn) to separate iPD from CN with an AUC of 0.91 and an accuracy of 86.2%. The high accuracy of this biomarker panel indicates that there may be a metabolite signature that could be used to assist in the diagnosis of iPD cases. Importantly, using samples from the AIBL study on AD, we demonstrated that the metabolite panel for iPD was specific for iPD compared to AD with the only overlapping metabolite being Cys. In AD, a panel of three metabolites (Asp, Cys, Trp), two ratios (Homoserine/*N*-Acetyl-phenylalanine, Pro/3-HK) and one interaction (Glu*Tryptamine) were able to separate AD from CN with an AUC of 0.88 and an accuracy of 79.3%. Future studies will be needed to validate the potential clinical impact of these diagnostic markers for iPD and AD.

**Fig. 2:**
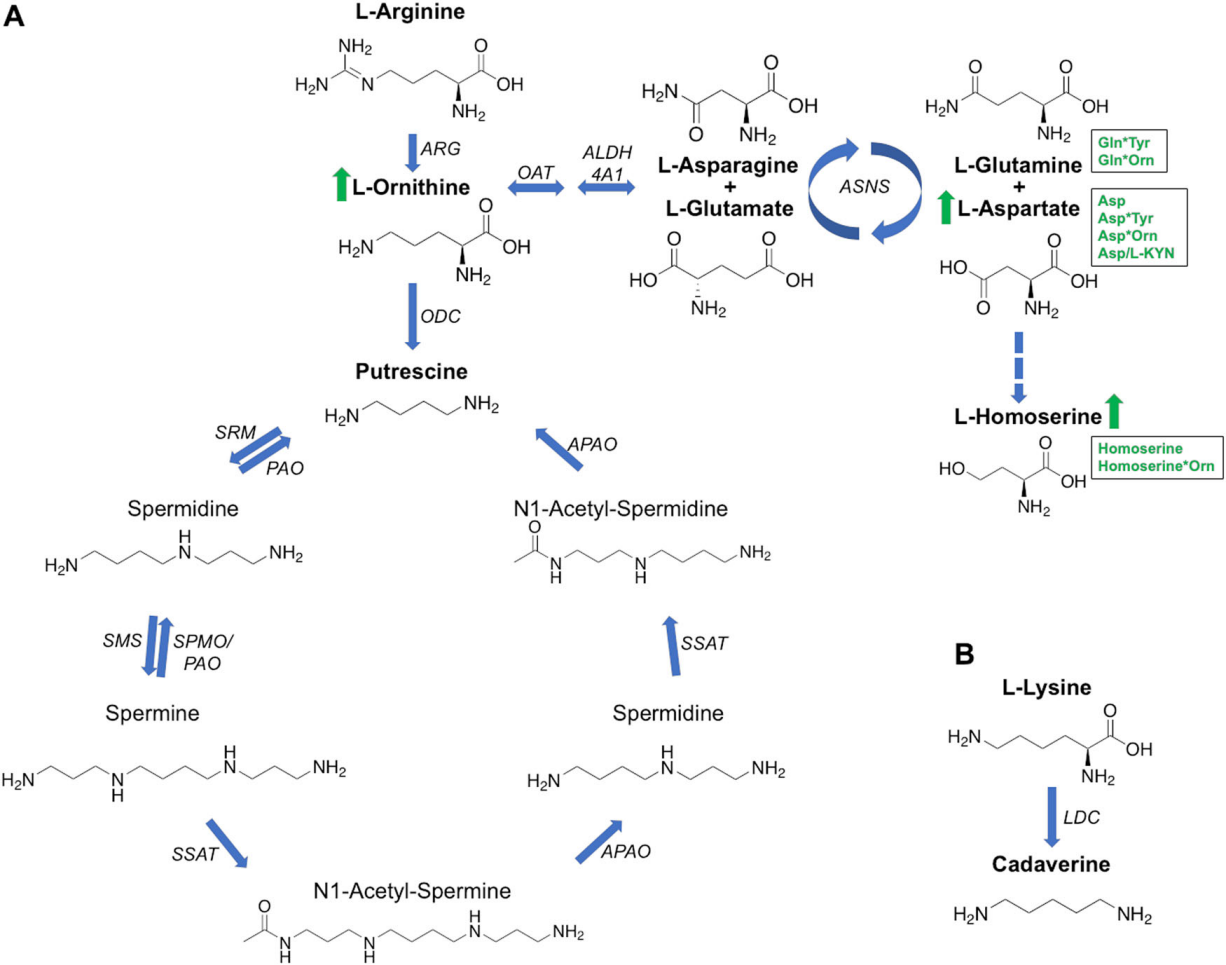
Polyamine pathway and Cadaverine pathway. **A** Polyamine pathway and **B** Cadaverine pathway. The metabolites highlighted in bold have been targeted and detected in this study. l-Ornithine, l-Glutamine and l-Aspartate are all significantly increased in the iPD cohort (arrows and text written in green). ARG arginase, ODC ornithine decarboxylase, SRM spermidine synthase, PAO polyamine oxidases, SMS spermine synthase, SPMO spermidine oxidase, SSAT1 and 2 diamine acetyltransferase 1 and 2, APAO acetylated polyamine oxidase, LDC lysine decarboxylase, OAT ornithine aminotransferase, ALDH4A1 delta-1-pyrroline-5-carboxylate dehydrogenase), ASNS asparagine synthetase [glutamine-hydrolysing]).

**Fig. 3:**
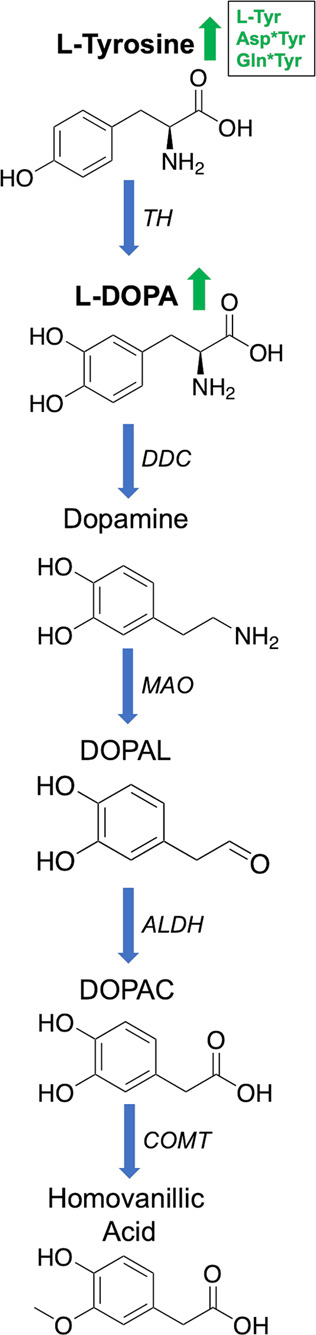
Dopamine-catecholaminergic pathway. The metabolites highlighted in bold have been targeted and detected in this study. Both Tyr and l-DOPA are significantly increased in iPD patients (arrows and text in green). TH tyrosine hydroxylases, DDC dopa decarboxylase, MOA monoamine oxidase, DOPAL 3,4-dihydroxyphenylacetaldehyde; toxic intermediate, ALDH aldehyde dehydrogenase, DOPAC 3,4-dihydroxyphenylacetic acid and COMT catechol-O-methyltransferase.

Looking at individual analyte concentrations, ratios and interactions, the most significant changes were observed for three metabolites of the KP (3-HK, l-KYN, 3-AA), and the amines of Asp, Orn, β-ala, Gln, Tyr, Homoserine and Cys. In the case of 3-HK, Orn and Tyr, all are increased in iPD compared to CN and AD and are therefore disease-specific. Asp was significantly increased in the serum of iPD patients (mean: 12.57 pmol µL^−1^) and slightly in naïve-iPD patients (mean: 10.81 pmol µL^−1^), when compared to CN group (mean: 9.69 pmol µL^−1^). In previous studies, changes in plasma Asp levels were inconsistent in iPD patients, as probably a reaction to the treatment^25,69^. However, as Asp is also involved in two interactions (Asp*Tyr, Asp*Orn), one ratio (Asp/l-KYN) and is also part of the iPD/CN separation model, it may have an important role in iPD. Further, Asp is converted into Homoserine by a two-reduction step of the terminal carboxyl group. Not surprisingly, both metabolites were increased in PD patients in this study (Fig. 2). The Asp-Homoserine intermediate is the branching point for the lysine pathway, and Homoserine itself is the metabolic branching point of threonine. Tyr was significantly increased in l-DOPA treated iPD patients (mean: 102.73 pmol µL^−1^) compared to CN (mean: 86.02 pmol µL^−1^). In drug-naïve patients, Tyr-increase was not significantly changed validating the lack of l-DOPA treatment and that the changes in Tyr are associated with the treatment (mean: 88.82 pmol µL^−1^). Tyr is converted to l-DOPA via tyrosine hydroxylases (TH1-4) and l-DOPA is the precursor of dopamine (Fig. 3)^70^. TH enzymes can also catalyze the hydroxylation of phenylalanine to Tyr^71^. TH enzymes are mainly expressed in dopaminergic neurons. In iPD, however, most dopaminergic neurons are dead due to dopamine deficiency. The reason for the Tyr increase in l-DOPA treated iPD patients is unclear and could be related to the use of peripheral decarboxylase inhibitors contained in Levodopa treatment and/or the metabolism of the individual’s gut microbiome^72^. Cys was decreased ca. 10% in both iPD and naïve-iPD participants as compared with CN participants, while it was increased ca. 20% in AD participants (Table 1). Some studies report an increase of cysteine in serum after l-DOPA intake^23^, others report a decrease in plasma^73,74^. A decrease of cysteine is thought to be a reaction to l-DOPA intake and an indication of increased glutathione (GSH) synthesis due to oxidative stress^73–75^. GSH itself acts as a redox buffer and antioxidant defence, and its homoeostasis dysregulation is believed to contribute to the progression of neurodegenerative diseases^76^. However, this does not explain why iPD participants have a lower cysteine concentration when compared to untreated participants.

**Fig. 4:**
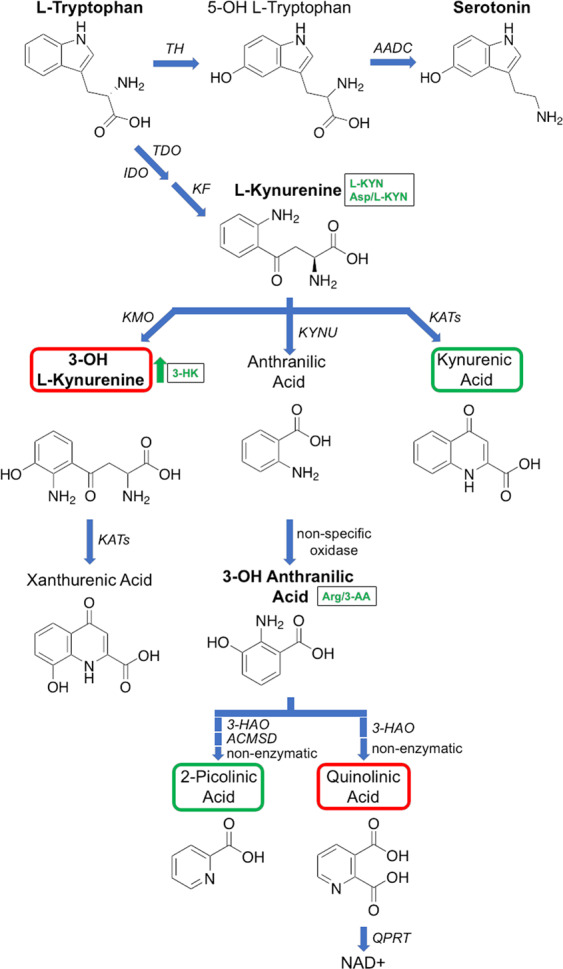
Tryptophan/kynurenine pathway. The main end product of this pathway is NAD+ (nicotinamide adenine dinucleotide). Trp can also be converted to serotonin. The metabolites highlighted in bold have been targeted and detected in this study. Metabolites surrounded by red boxes are neurotoxic and by green boxes are neuroprotective. l-Kynurenine, 3-OH l-Kynurenine and 3-OH anthranilic acid are all significantly changed in the iPD cohort (arrow and text written in green). IDO indoleamine 2,3-dioxygenase), TDO tryptophan 2,3-dioxygenase), KF kynurenine formamidase, KMO kynurenine 3-monooxygenase, KYNU kynureninase, KAT kynurenine aminotransferase, 3-HAO 3-hydroxyanthranilic acid dioxygenase, ACMSD 2-amino-3-carboxymuconate-6-semialdehyde decarboxylase, QPRT nicotinate-nucleotide pyrophosphorylase, TH tryptophan hydroxylase and AADC aromatic amino acid decarboxylase.

The KP is the central route in the Trp metabolism, ~95% of Trp is catabolized via the KP, leading to the formation of nicotinamide adenine dinucleotide and its phosphate (NAD + /NADP; Fig. 4)^77,78^. The remainder forms a substrate for serotonin and melatonin synthesis. Trp and KP metabolites have been studied since the early 1930s^79,80^. Altered KP metabolism is involved in a number of neurodegenerative diseases (e.g. Epilepsy^81^, Huntington’s disease^82^, Multiple sclerosis^77,83^, Amyotrophic lateral sclerosis^51^ and AD^84^). Their role in iPD has been known since the early 1990s^17^. This pathway is highly regulated, with small changes substantially disturbing its equilibrium^61^. l-KYN is the central metabolite of this pathway and is either degraded into kynurenic acid (KYNA), 3-HK or anthranilic acid (AA) (Fig. 4)^19^. In the central nervous system, ~ 40 % of l-KYN is locally produced, whereas the other 60 % are absorbed from blood^85^. It can be transported across the blood–brain barrier (BBB) by a neutral amino acid carrier^86^, which is thought to be modulated by l-valine in metabolic disorders. KYNA acts as a neuroprotectant and could therefore have therapeutic effects in neurological disorders like iPD^54,55^, but its use is restricted due to its very limited ability to cross the BBB^86^. 3-HK and AA have been shown to cause neuronal damage, as they generate free radicals and elevate oxidative stress^19,58^. 3-AA, synthesised from 3-HK and/or AA, exhibits an increased level in iPD patients^26^. Another metabolite of the KP pathway is quinolinic acid (QUIN), acting as an excitotoxic agonist of the NMDA receptor^87^. In contrast, KYNA has been shown to protect rat neurons against the damage caused by QUIN^88,89^. NAD + /NADP is one of the final products of the KP, produced by the catabolism of QUIN. Monocytes can be activated by high levels of inflammatory cytokines, which upregulate the expression of KP enzymes, favouring the production and secretion of QUIN^90^. Furthermore, QUIN has been shown to induce damage to dendrites and axons, when present in high/toxic levels, leading to cytoskeleton destabilization by the phosphorylation of structural proteins^91,92^. In this study, l-KYN was significantly decreased in the serum of iPD patients (iPD 2.59 vs CN 3.02 pmol µL^−1^, mean values), whereas 3-HK was increased (iPD 0.05 vs CN 0.04 pmol µL^−1^, mean values) (Table 1). Our results are in line with other studies^19,22,93,94^. Further, we observed a decrease in the ratios of l-KYN/Trp (CN: 0.04; iPD: 0.035), l-KYN/3-HK (CN: 75.5; iPD: 51.8) and Arg/3-AA (CN: 2037.5; iPD: 1845.3) in iPD patients, and an increase of Asp/l-KYN (CN: 3.21; iPD: 4.85) (Table 1). Importantly, we show that the changes were also detected in naïve-iPD patients (l-KYN/Trp: 0.031; KYN/3-HK: 49; Asp/l-KYN: 4.41), indicating that the concentration changes of l-KYN and 3-HK are based on iPD and not on l-DOPA treatment. A decrease of l-KYN/3-HK is associated with an increased kynurenine 3-monooxygenase (KMO) activity^32^. The KMO enzyme catalyses the hydroxylation of l-KYN to form 3-HK. Inhibition of the KMO enzyme has been shown to reduce LID in Parkinson’s like disease in monkeys^95^. Several reviews about the KP pathway and its role in the central nervous system are available^54,96–98^. It is highly recommended that future studies should simultaneously analyse all relevant KP metabolites (Trp, l-KYN, 3-HK, xanthurenic acid, AA, 3-AA, QUIN, KYNA, 2-picolinic acid and NAD + /NADP; Fig. 4) as their function can be either neuroprotective or neurotoxic. It is unclear at this point, if the metabolite changes found in serum are originating from the brain, or if they influence brain homoeostasis.

PAs are small aliphatic polycations that are derived from the amino acids Met, Orn, Arg and Lys^99^. The most common PA are spermine, spermidine, Put and Cad (Fig. 2). When the PA metabolism is disturbed, multiple cellular processes are influenced (e.g. gene expression, protein translation, autophagy and membrane function^59,99^). PA have been associated with neurodegenerative diseases (e.g. AD^100^, Amyotrophic lateral sclerosis^27^ and iPD^101^). In iPD patients, the metabolites Cad and Put were increased^21,102^, as well as Orn^103^ and the Put/Orn ratio^104^. PA were also found to be increased in astroglial cells^105^. Cellular PA also promotes the aggregation and fibrillization of α-synuclein, which is the major protein component of Lewy bodies in iPD^106^. However, it is thought, albeit controversially, that PA may be neuroprotective as they can induce autophagy as a way to protect cells from stress^56,57^ and have been shown to be elevated in neurodegenerative diseases^27,100^. Additionally, they have been found to be cytotoxic, with their increase leading to an elevated concentration of toxic metabolites such as aldehydes and hydrogen peroxide^59,60^. PA can also have several opposing effects on NMDA receptors, including a glycine-dependent potentiation, a voltage-dependent inhibition and a voltage- and glycine-independent potentiation^3,107^. For example, spermine can bind to NMDA receptor and potentiate agonist-induced currents^108^. Finally, the increase of PA in iPD and AD brain could also be based on the level of their enzymes increasing as a reaction to proteasomal impairment^109^. Although we did not find any significant changes in Cad, Put and Put/Orn ratio after confounder adjustment, its precursor Orn was significantly increased in iPD patients (iPD 74.95 vs CN 63.82 pmol µL^−1^, mean values), involved in four interactions (Homoserine*Orn, β-ala*Orn, Asp*Orn and Gln*Orn) and also part of the iPD/CN separation model. Reiterating the above, future studies should aim to include all possible PAs, in order to gain a better understanding of the role of this highly relevant and regulated pathway.

Both kynurenines and PAs can bind to the NMDA receptors^62^, indicating that this ion channel complex is a potential therapeutic target. In particular, spermine was shown to attenuate or prevent QUIN-induced damages in rat striatum through NMDA receptor interaction and/or its antioxidant function^110^. Spermidine was also shown to be neuroprotective against QUIN-induced excitotoxic cell death due to its NMDA receptor antagonistic properties^111^. Therefore, a common denominator of both pathways are NMDA receptors. Moreover, it seems to be crucial that both pathways need to be in perfect balance to guarantee normal cellular function. Neither spermine, spermidine nor Quin were part of this study but should be included in future studies due to their significant biological importance.

To get an overview of already known metabolic changes in iPD patients in regard to proteinogenic amino acids and metabolites of the kynurenine and polyamine pathways, we performed a literature review in PubMed (details outlined in method section). In total, we identified 32 cohort studies, comparing iPD with CN and/or AD, restless leg syndrome (RLS), traumatic brain injury (TBI), multiple system atrophy (MSA), Amyotrophic lateral sclerosis, Huntington’s Disease and progressive supranuclear palsy (PSP). From that 32 studies, 12 analysed metabolites of the KP (Table 3A)^17,19,22–24,26,29,31,32,93,112,113^, 18 analysed amino acids (Table 3B)^20,23,25,29,43,50–53,69,73,112,114–119^ and five PA (Table 3C)^18,21,27,102,120^. Like in this study, eight studies also analysed the blood serum of iPD patients, whereof five covered amino acids and another five metabolites of the KP. In the remaining studies, metabolites were extracted and analysed from the brain, plasma, CSF, urine and RBC. Only the main and significant changes between iPD and CN are shown. A red arrow (↓) indicates a decrease in the metabolite concentration in iPD, a green arrow (↑) an increase, a yellow one (⟷) no changes and an empty box indicates nonsignificant changes/non-analysed metabolites. Moreover, two stoichiometric/pathway enrichment analyses were also included. The pathway enrichment analysis was performed by Kori M. et al. (2016) and 54 metabolite biomarkers were proposed for iPD, including many proteinogenic amino acids^119^. However, many studies led to controversial results where the same bio-fluid was analysed; and where the same metabolites had discorded results. A possible explanation is the sample size of the analysed cohorts, with large variance within biomarkers across groups resulting in nonsignificant increases or decreases in the same biomarkers. In 43 %, the cohort size was ≤50 participants and 73% of all studies had ≤100 participants. Only three studies have a cohort size of >200, including ours.

**Table 3:** Literature review of metabolite changes in PD patients compared to control, including (A) metabolites of the kynurenine pathway, (B) proteinogenic amino acids and (C) metabolites of the polyamine pathway.

No	Study	Research focus	Biomaterial	Cohort size (*n*)	Screening technique	Metabolites						Metabolite ratios				
						Trp	KYN	3-HK	AA	KYNA	QUIN	3-HK/ KYNA	KYN/Trp	KYN/3-HK	KYN/KYNA	KYNA/QUIN
(A) Kynurenine pathway metabolites																
0	This study	PD vs. CN/AD	Serum	231	LC-MS		↓	↑					↓	↓		
1	Widner et al., 2002^93^	PD vs. CN	Serum + CSF	33	HPLC	↓	↓						↑			
2	Schulte et al., 2016^23^	PD vs. RLS/CN	Serum	1449	LC-MS + GC-MS	**⟷**	**⟷**									
3	Hatano et al., 2016^31^	PD vs. CN	Serum	50	LC-MS + GC-MS	↓										
4	Han et al., 2017^22^	PD vs. CN	Serum	85	LC-MS	↓	↓	↑								
5	Sorgdrager et al., 2019^112^	PD vs. AD/CN	Serum & CSF	105	LC-MS	↓		↓		↓			**⟷**			↓
6	Oxenkrug et al., 2017^24^	PD vs. AD/CN	Plasma	62	LC-MS	↓	↑		↑	↑			↑			
7	LeWitt et al., 2013^19^	PD vs. CN	CSF	105	LC-MS + GC-MS			↑				↑				
8	Iwaoka et al., 2020^113^	PD vs. CN	CSF	33	HPLC ECD		↑	↑								
9	Havelund et al., 2017^68^	PD vs. CN	Plasma & CSF	40	LC-MS				↓			↑		↓		
10	Hartai et al., 2005^83^	PD vs. CN	Plasma & RBC	36	HPLC					↓ plasma ↑ RBC						
11	Ogawa et al., 1992^17^	PD vs. CN	Brain regions	−85	HPLC ECD		↓	↑		↓			**⟷**	↓	**⟷**	
**12**	Luan et al., 2015^47^	PD vs. CN	Urine	157	LC-MS + GC-MS	↑	↑						↑			

No	Study	Research focus	Biomaterial	Cohort size (*n*)	Screening technique	Arg	His	Lys	Asp	Glu	Ser	Thr	Asn	Gln	Cys	Sec	Gly	Pro	Ala	Val	Ile	Leu	Met	Phe	Tyr	Trp
(B) Amino acids																										
0	This study	PD vs. CN/AD	Serum	231	LC-MS				↑	↑															↑	
1	Hirayama M. et al., 2016^53^	PD vs. CN	Serum & sweat	58 & 107	LC-MS + HPLC fluorescence																			↑	↓	
2	Schulte et al., 2016^23^	PD vs. CN/RLS	Serum	1449	LC-MS + GC-MS	↓			↓	↓	↓	↑	↑	↑	↑		↑					↓		↓	↑	**⟷**
3	Fiandaca M. S. et al., 2018^114^	PD vs. CN/TBI	Serum	155	LC-MS					↓																
4	Figura M. et al., 2018^52^	ePD, lPD + /− LID (no CN)	Serum	73	HPLC fluorescence	↓						↑							↓					↓		
5	Sorgdrager et al., 2019^112^	PD vs. CN/AD	Serum & CSF	105	LC-MS															↓		↓		↓	↓	↓
6	Iwasaki Y. et al., 1992^25^	PD vs. CN	Plasma	40	Ion exchange				↑	↑							↑									
7	Mueller T. et al., 2012^73^	l-Dopa & PD (no CN)	Plasma	13	HPLC ECD										↓											
8	Kuiper M.A. et al., 2000^50^	PD vs. CN/AD/MSA	CSF	156	HPLC fluorescence	↑																				
9	Engelborghs S. et al., 2003^115^	PD vs. CN	CSF	54	HPLC ECD	- No significant changes detected (Asn,Cys, Sec, Pro and Trp not included) **-**																				
10	Oehman A. et al., 2015^20^	PD vs. CN	CSF	20	^1^H-NMR spectr.														↓					↓		
11	Jimenez-Jimenez F. J. et al., 1996^69^	PD vs. CN	CSF & plasma	76	Ion exchange				↓				↑	↑			↑									
12	Molina J.A. et al., 1997^116^	PD vs. CN	CSF & plasma	76	Ion exchange							↑								↓		↓	↑		↑	↓
13	Trupp et al., 2014^43^	PD vs. CN	CSF & plasma	40	GC-MS						↑								↑				↑			↓
14	Wuolikainen et al., 2016^51^	PD vs. CN/ALS	CSF & plasma	72	LC-MS + GC-MS		↑					↑		↑					↑		↑	↑				
15	Mally J., et al., 1997^117^	PD vs. CN	CSF & serum	20	HPLC fluorescence					↓				↑												
16	Luan et al., 2015^47^	PD vs. CN	Urine	157	LC-MS + GC-MS			↑									↑	↑	↑		↑	↑		↑	↑	↑
17	Sertbas et al., 2014^118^	PD vs. CN	/	/	Stoichiometric model						(+)						(+)		(+)	(+)	(+)	(+)	(+)			
18	Kori M. et al., 2016^119^	PD vs. AD/ALS	/	/	Pathway enrichment	(+)			(+)	(+)				(+)				(+)	(+)							

No	Study	Research focus	Biomaterial	Cohort Size (*n*)	Screening technique	Cad	Put	Spd	Spm
(C) Polyamines									
0	This study	PD vs. CN/AD	Serum	231	LC-MS				
1	Saiki et al., 2019^120^	PD vs. CN/AD	Plasma	467	LC-MS + CE-MS			↑	↓
2	Paik M-J. et al., 2010^21^	PD vs. CN/MSA	CSF	42	GC-MS	↑	↑	↓	
3	Gomes-Trolin C. et al., 2002^27^	PD vs. CN/ALS	RBC	60	HPLC fluorescence		↓	↑	↑
4	Betancourt L. et al., 2018^102^	PD vs. CN	RBC	24	LC-MS		↑		
5	Vivo M. et al., 2001^18^	PD vs. CN/HD/PSP	Brain	48	HPLC fluorescence		no difference to CN		

The following parameters are shown: Study origin, research focus, source of biomaterial, total cohort size, screening technique, metabolite changes (↓ decrease, ↑ increase, ⟷ no change) and metabolite-ratio changes. The gaps in the table refer to non-analyzed or nonsignificantly changed metabolites.

iPD and its progression seem to lead to global metabolic changes not only in the brain but also in peripheral body fluids. Overall, our study adds to the body of evidence that amino acids and metabolites of the KP are changed in patients with iPD. Most above-mentioned studies, including our own, did not collect information about the diet of the participants (e.g. western diet, ketogenic diet, vegan, etc.). However, it is well known that the diet and the gut microflora have a significant impact on the metabolome^121–124^. In particular, the microbiome has been documented to alter the metabolic profile and activity in humans^125^. Therefore, the impact of the diet on the results cannot be excluded, and this is a limitation of this study.

Taking all these changes into consideration, the detailed molecular mechanism of iPD is still poorly defined. Therefore, a panel of biomarker is urgently needed to increase iPD diagnosis and treatment success. Nevertheless, serum metabolomics is a powerful tool for the discovery and development of a blood-based small-molecule biomarker for neurodegenerative diseases like PD.

## Methods

### Patient recruitment and cohort details

In the present study, a targeted metabolite screen was performed on provided blood sera from 231 clinically assessed individuals, collected as previously described^126^. All samples were processed and stored under identical conditions; at −178 °C in liquid nitrogen dewars as previously described^127^. Eighty-eight serum samples were taken from the Australian Imaging, Biomarker & Lifestyle Flagship Study of Ageing (AIBL; 60 healthy age-matched controls with 29 males and 31 females and 28 patients with diagnosed AD including 8 males and 20 females). About 143 samples were taken from the Australian Parkinson’s Disease Registry (APDR; 33 healthy age-matched controls with 20 males and 13 females, 103 patients with l-DOPA treated iPD including 65 males and 38 females and 7 drug-naïve iPD patients with 6 males and 1 female). The demographic and clinical features of the analysed cohort are summarised in Table 4.

**Table 4:** (A) Demographic and (B) clinical features of an analyzed cohort.

	iPD (*n* = 103)		drug-naïve iPD (*n* = 7)		Control (CN) (*n* = 93)		AD (*n* = 28)		*n* (Sum)
	Male	Female	Male	Female	Male	Female	Male	Female	
(A) Demographic features of analysed cohort									
*n*	65	38	6	1	49	44	8	20	231
Median age years (SD)	65 (9.7)	67.5 (7.9)	66.5 (7.8)	48 (−)	76 (9.5)	74 (8.6)	75.5 (7.9)	73 (8.5)	NA

	Age at assessment	Age at onset of PD symptoms	Duration of disease	Total levodopa equivalent	MDS-UPRDS I	MDS-UPDRS II	MDS-UPRDS III	MDS-Part IV	MDS-UPDRS total	Schwab & England	Hohn & Yahr
(B) Clinical features of analysed cohort											
Number of values	104	104	104	103	103	103	104	104	104	104	104
Minimum	41	29	0	0	1	1	0	0	11	0	0
25% Percentile	60	52	3	450	7	5	12	0	31	80	2
Median	66	58	6	750	10	8	20	3	43.5	90	2
75% Percentile	71	63.8	10	1178	15	14	32.8	6	61	90	2
Maximum	87	80	31	3351	31	31	68	17	136	100	5
Range	46	51	31	3351	30	30	68	17	125	100	5
10% Percentile	54	43	2	40	4.4	3	7	0	22	75	1
90% Percentile	75	71.5	13	1500	18	19	43	9.5	80.5	100	3
95% CI of median											
Lower confidence limit	63	56	5	675	9	7	16	2	37	90	2
Upper confidence limit	68	61	8	900	13	10	24	4	52	90	2
Mean	65	57.9	7.1	868.1	11.3	10.1	22.9	3.7	47.8	84.6	2
Std. deviation	8.9	10.1	5.2	632.9	5.8	6.4	14.2	3.8	23.7	14.1	0.8
Std. errror of mean	0.9	1	0.5	62.4	0.6	0.6	1.4	0.4	2.3	1.4	0.1
Lower 95% CI of mean	63.3	55.9	6.1	744.4	10.2	8.8	20.2	3	43.2	81.9	1.8
Upper 95% CI of mean	66.7	59.9	8.1	991.8	12.4	11.3	25.7	4.5	52.4	87.4	2.1

### Ethics statement

For both cohorts, experiments were conducted under The University of Melbourne human ethics committee approval ID1136882. All participants provided written informed consent prior to enrolment.

### Chemicals

The following chemicals were used in this study: Acetonitrile (AcN) (Sigma A955-4), boric acid (Sigma B0394-500G), 6-aminoquinolyl-*N*-hydroxysuccinimidyl carbamate (AQC, Synchem UG & Co KG S041), formic acid (FA) (Sigma 27001-500ML-R). Amino acid standards were purchased from Sigma Aldrich at a minimum purity of ≥99%.

### Sample preparation and derivatization

Metabolites were extracted and derivatized as previously reported^64^. In detail, 50 μL of human serum was transferred into a 1.5 mL Eppendorf tube. Next, 150 μL of ice-cold methanol containing an internal standard (ISTD, ^13^C_5_,^15^N-l-Valine, Sigma Aldrich, 25 µM) was added. All samples were vortexed and cooled on wet ice for 30 min. Samples were then centrifuged at 15,000 × *g* (maximum speed) for 10 min to precipitate protein, and the supernatant was transferred to a clean, 1.5 mL Eppendorf tube. Two separate sample concentrations were prepared, an undiluted sample and a 5× concentrated sample. The undiluted sample, 10 µL of the supernatant was derivatized directly as described below. For the concentrated sample, 50 µL of each supernatant was dried down and reconstituted in 10 µL of 75% methanol (MeOH), 0.1% FA to form a fivefold concentration ready for derivatization. In addition to this, a pooled biological quality control (PBQC) was prepared to monitor the performance of the 6410 and 6490 QQQ LC/MS instruments. For derivatization, 2.85 mg of AQC was dissolved in 1 mL anhydrous AcN. Next, 70 μL of borate buffer (pH 8.8) was added to 10 μL of each sample. The samples were vortexed and centrifuged at 15,000x*g* for 1 min. Next, 20 μL of AQC solution was added and the samples were vortexed and centrifuged (1 min, maximum speed) again, followed by a 10 min incubation step at 55 ˚C. The samples were then vortexed and centrifuged at maximum speed for 10 min. Finally, 20 μL of each sample was transferred to a glass vial for analysis on the 6410 and 6490 QQQ LC/MS instruments.

### Mass spectrometry instrumentation

Serum extracts were separated on an Agilent 1200 LC-system using an Agilent ZORBAX Eclipse PLUS C18 column (2.1 mm × 50 mm, 1.8 µM, Product number 959757-902). Elution was carried out with a water/AcN mobile phase binary solvent system. Mobile phase A consisted of 100% water/0.1% FA; mobile phase B consisted of 100% AcN/0.1% FA. The samples were analyzed by Agilent 6410 and 6490 ESI-QQQ-MS instruments (Santa Clara, CA) in dynamic multiple reaction monitoring (dMRM) positive ionisation mode using the same instrument settings and method as previously described^64^.

### Selected metabolites

In total, we included 38 metabolites in our targeted screen. Each metabolite was identified based on a standard, which were all purchased from Sigma Aldrich. They represent several different pathways, including the cysteine pathway, the phenylalanine/tyrosine/l-DOPA pathway, the polyamine pathway and the tryptophan/kynurenine catabolic pathway. The following 20 proteinogenic amino acids were included: l-Arginine (Arg), l-Histidine (His), l-Lysine (Lys), l-Aspartic Acid (Asp), l-Glutamic Acid (Glu), l-Serine (Ser), l-Threonine (Thr), l-Asparagine (Asn), l-Glutamine (Gln), l-Cysteine (Cys), Glycine (Gly), l-Proline (Pro), l-Alanine (Ala), l-Valine (Val), l-Isoleucine (Ile), l-Leucine (Leu), l-Methionine (Met), l-Phenylalanine (Phe), l-Tyrosine (Tyr) and l-Tryptophan (Trp). The other metabolites included were: the two PAs of cadaverine-2 (Cad) and putrescine-2 (Put) and l-3,4-dihydroxyphenylalanine (l-DOPA), serotonin (sero), l-KYN, 3-HK, 3-AA, 4-OH-Pro, homoserine, β-ala, *N*-acetyl-phenylalanine, tryptamine, Orn, citrulline, Sarco, γ-aminobutyric acid (GABA), 2-Ambut and taurine (Tau). Quantitation was conducted by constructing an external standard curve as described previously^64^. The chemical structures of the metabolites have been drawn with ChemDraw JS online (https://chemdrawdirect.perkinelmer.cloud/js/sample/index.html#).

### Statistical analyses

Metabolite and amino acid biomarker data were cleaned via interquartile range filtering and log-transformed prior to analyses. Means and SD are presented post interquartile range filtering (Table 1). Age and gender effects were tested via Pearson’s correlation and independent samples *t*-test (Supplementary Table 2). Interactions between disease state (Control (CN) vs iPD participants) and age/gender were tested for each metabolite/amino acid via GLM to determine whether either confounder had a significant effect on metabolite via disease state (Supplementary Table 2).

Statistical analyses of metabolites and amino acids was set in two separate hypotheses; (1) targeted assessment of 37 known biomarkers (excluding l-DOPA) using nominal significance (Table 1) and (2) discovery of ratio’s and interactions between all possible metabolites and amino acids using a Bonferroni adjusted alpha (α = 0.05/K ratio’s and interactions, Supplementary Tables 3 and 4). For both hypotheses, independent samples *t*-test was used to test mean analyte levels between all groups (CN vs iPD, CN vs drug-naïve iPD and CN vs AD), and where the sample size was large enough (i.e. not including the drug-naïve participants) a GLM was used to account for age and gender. Disease specificity was tested across the 37 analytes between iPD and AD groups (targeted assessment using independent samples *t*-test, Table 1 and discovery using GLM, Supplementary Tables 3 and 4). The final number of ratios and interactions was reduced via the removal of those with an SD of less than 0.5.

Multivariate modelling to find an optimal set of metabolites associated with outcome was performed using both the least absolute shrinkage and selection operator (LASSO) followed by model selection to reduce the possibility of over fitting. Both individual metabolite biomarkers and selected sets of biomarkers from the multivariate testing were then tested using ROC analyses. Multivariate and ROC analyses were performed using the R statistical environment^128^ (https://www.R-project.org/). Biomarker significance was retained using Bonferroni correction to account for multiple testing.

### Literature review

The 32 cohort studies presented in Table 3 were selected the following way: We searched PubMed for publications with the terms ‘PD’ AND ‘Kynurenines’ or ‘Amino Acids’ or ‘PAs’. Only significant changes for PD are shown.

### Reporting Summary

Further information on research design is available in the Nature Research Reporting Summary linked to this article.

## Supplementary information

Supplementary Information

Reporting Summary

## Data Availability

Anonymized data will be shared on request from any qualified investigator for purposes of replicating procedures and results.
