# Supplementary material for: A six-metabolite panel as potential blood-based biomarkers for Parkinson’s disease
Source: NPJ Parkinsons Dis. 2021 Oct 14;7:94. doi: 10.1038/s41531-021-00239-x (PMC8516864; doi:10.1038/s41531-021-00239-x)
Supplement: Supplementary file 1 — Supplementary Information [file 41531_2021_239_MOESM1_ESM.pdf]

Supplement of:

A six-metabolite panel as potential blood-based biomarkers for Parkinson's disease.

Stephan Klatt[1,2], James D. Doecke[2,3], Anne Roberts[1,2], Berin A. Boughton[4,5], Colin L. Masters[1,2], Malcolm Horne[1], Blaine R. Roberts*[6,7]

Supplementary Table 1. Mean metabolite concentrations (pmol*µL^{-1}) with standard deviations (SD) and results of pairwise comparison prior and after the adjustment for age and sex. Additional data to Table 1.

Metabolite	Mean (SD); in picomoles/µL				Unadjusted for confounders p-values			Adjusted for confounders p-values
	CN (n = 93)	naïve-iPD (n = 7)	iPD (n = 103)	AD (n = 28)	naïve-iPD vs iPD	naïve-iPD vs CN	iPD vs AD	iPD vs AD
**2-Ambut**	22.03 (6.72)	24.34 (9.77)	20.7 (5.33)	20.49 (8.59)	0.37	0.51	0.38	0.27
**3-AA**	0.04 (0.01)	0.03 (0.01)	0.04 (0.02)	0.03 (0.01)	0.55	0.32	**6.20E-05**	0.002
**3-HK**	0.04 (0.02)	0.05 (0.06)	0.05 (0.04)	0.03 (0.01)	0.22	0.98	**0**	**1.05E-03**
**4-OH-Pro**	11.37 (5.01)	13.97 (6.83)	12.16 (4.94)	12.41 (5.35)	0.48	0.28	0.93	0.51
**Ala**	414.07 (81.01)	438.91 (119.44)	432.91 (90.87)	407.2 (80.2)	0.96	0.67	0.18	0.02
**Arg**	81.5 (20.04)	76.93 (24.66)	73.81 (15.54)	85.11 (19.45)	0.97	0.56	0.02	0.27
**Asn**	41.65 (6.23)	42.59 (7.95)	42.59 (6.61)	38.65 (4.35)	0.91	0.81	**4.46E-04**	0.01
**Asp**	9.69 (5.32)	10.81 (5.71)	12.57 (4.99)	9.83 (3.82)	0.42	0.96	0.02	0.002
**β-ala**	4.52 (1.59)	4.69 (1.96)	5.19 (1.64)	4.41 (1.8)	0.41	0.85	0.02	0.01
**Cad**	0.2 (0.09)	0.13 (0.07)	0.17 (0.08)	0.22 (0.1)	0.25	0.04	0.002	0.01
**Citrulline**	34.61 (8.12)	32.03 (7.7)	33.06 (7.22)	37.06 (9.57)	0.67	0.41	0.10	0.81
**Cys**	177.14 (28.59)	160.55 (29.36)	157.78 (27.56)	191.13 (22.99)	0.84	0.23	**0**	**9.83E-04**
**GABA**	0.32 (0.17)	0.34 (0.19)	0.31 (0.17)	0.35 (0.16)	0.56	0.75	0.11	0.94
**Gln**	710.1 (81.81)	729.43 (140.56)	690.75 (80.77)	711.35 (65.59)	0.58	0.81	0.17	0.19
**Glu**	45.94 (20.46)	52.99 (13.61)	54.88 (20.47)	38.74 (15.61)	0.91	0.12	**7.40E-05**	**1.76E-04**
**Gly**	274.57 (67.96)	268.57 (63.31)	298.52 (86.63)	294.61 (61.72)	0.38	0.84	0.49	0.69
**His**	70.47 (12.75)	75.28 (8.27)	70.58 (11.31)	69.25 (11.27)	0.10	0.10	0.60	0.87
**Homoserine**	0.37 (0.14)	0.42 (0.11)	0.43 (0.13)	0.42 (0.15)	0.90	0.12	0.50	0.32
**Ile**	78.89 (14.87)	81.76 (11.6)	83.54 (17.3)	74.58 (19.6)	0.72	0.64	0.01	0.05
**L-KYN**	3.02 (0.78)	2.45 (0.7)	2.59 (0.7)	2.69 (0.94)	0.58	0.09	0.83	0.64
**Leu**	156.23 (28.16)	167.94 (29.47)	165.33 (34.58)	139.5 (25.99)	0.31	0.18	**1.76E-04**	0.01
**Lys**	229.23 (33.62)	219.13 (10.11)	222.4 (36.23)	214.97 (35.57)	0.72	0.15	0.26	0.05
**Met**	29.94 (4.36)	30.21 (3.13)	30.62 (5.23)	27.73 (4.29)	0.75	0.83	0.005	0.04
**N-acetyl-phenylalanine**	0.03 (0.01)	0.02 (0)	0.03 (0.01)	0.02 (0.01)	0.75	0.71	**2.30E-05**	0.0016
**Orn**	63.82 (16.44)	66.93 (26.17)	74.95 (16.14)	61.41 (12.1)	0.32	0.88	**7.30E-05**	**3.03E-04**
**Phe**	79.92 (9.29)	80.7 (10.2)	83.44 (11.08)	75.63 (8.83)	0.48	0.85	**1.75E-04**	0.002
**Pro**	228.05 (54.83)	274.65 (98.72)	246.8 (76.35)	259.25 (83.25)	0.43	0.22	0.49	0.42
**Put**	0.28 (0.15)	0.23 (0.07)	0.24 (0.18)	0.29 (0.15)	0.42	0.44	0.02	0.06
**Sarco**	1.8 (0.63)	2.08 (0.89)	1.83 (0.61)	1.7 (0.66)	0.60	0.59	0.23	0.52
**Ser**	89.3 (17.26)	86.46 (16.05)	92.55 (18.43)	91.36 (16.96)	0.96	0.70	0.69	0.61
**Serotonin**	0.39 (0.22)	0.34 (0.21)	0.43 (0.32)	0.33 (0.24)	0.72	0.62	0.14	0.22
**Tau**	111.79 (31.34)	124.97 (35.26)	124.91 (32.11)	121.06 (28.68)	0.80	0.49	0.32	0.09
**Thr**	125.32 (23.7)	122.05 (20.64)	137.59 (31.11)	122.77 (26.25)	0.15	0.77	0.02	0.06
**Trp**	75.04 (13.19)	78.18 (10.4)	74.46 (12.72)	65.49 (12.27)	0.19	0.20	0.002	0.07
**Tryptamine**	0.02 (0.01)	0.02 (0.01)	0.02 (0.01)	0.02 (0.01)	0.92	0.91	0.01	0.02
**Tyr**	86.02 (14.66)	88.82 (7.86)	102.73 (25.51)	78.74 (15.1)	0.01	0.28	**0**	**8.40E-05**
**Val**	276.45 (47.38)	292.45 (31.63)	283.03 (53.43)	248.16 (46.45)	0.20	0.13	**1.40E-03**	0.02

Supplementary Table 2. Age and gender effects of tested cohort. Possible effects were tested via Pearson's correlation and Independent Samples t-test. Interactions between disease state (CN vs iPD participants) and age/gender were tested for each metabolite/amino acid via Generalized Linear Modelling (GLM) to determine whether either confounder had a significant effect on metabolite via disease state.

Metabolite	Mean (SD) Males	Mean (SD) Females	T-test p-value	Pearson's R correlation coefficient with age	p-value	Interaction CN/iPD*Age p-value	Interaction CN/iPD*Sex p-value
Ala	6.03 (0.21)	6.02 (0.19)	0.683	0.029	0.664	0.181	0.266
2-Ambut	3.02 (0.28)	3 (0.3)	0.600	-0.044	0.507	0.005	0.444
Arg	4.32 (0.23)	4.36 (0.22)	0.225	0.253	<0.001	0.675	0.417
Asn	3.75 (0.13)	3.68 (0.14)	<0.001	-0.094	0.155	0.463	0.022
Asp	2.22 (0.57)	2.36 (0.46)	0.034	-0.120	0.069	0.449	0.715
β-ala	1.59 (0.3)	1.4 (0.36)	<0.001	0.043	0.518	0.095	0.006
Cad	-1.78 (0.45)	-1.78 (0.51)	0.977	0.221	<0.001	0.272	0.576
Citrulline	3.5 (0.22)	3.52 (0.23)	0.473	0.319	<0.001	0.799	0.969
Cys	5.1 (0.19)	5.14 (0.18)	0.145	0.500	<0.001	0.158	0.915
GABA	-1.31 (0.57)	-1.23 (0.57)	0.329	0.186	0.005	0.619	0.754
Gln	6.56 (0.11)	6.53 (0.11)	0.018	0.084	0.202	0.483	0.076
Glu	3.85 (0.42)	3.77 (0.41)	0.178	-0.120	0.070	0.045	0.960
Gly	5.59 (0.2)	5.68 (0.29)	0.010	0.049	0.460	0.659	0.255
His	4.27 (0.15)	4.21 (0.18)	0.009	-0.095	0.151	0.477	0.012
Homoserine	-0.96 (0.37)	-1.01 (0.4)	0.414	-0.037	0.578	0.291	0.684
3-AA	-3.27 (0.43)	-3.49 (0.44)	<0.001	-0.130	0.048	0.438	0.396
4-OH-Pro	2.47 (0.39)	2.3 (0.39)	<0.001	0.072	0.276	0.037	0.203
3-HK	-3.36 (0.54)	-3.46 (0.57)	0.184	-0.116	0.080	0.078	0.659
Ile	4.43 (0.2)	4.29 (0.19)	<0.001	-0.115	0.080	0.964	0.239
Kyn	1 (0.27)	0.97 (0.28)	0.426	0.306	<0.001	0.060	0.124
Leu	5.11 (0.19)	4.97 (0.17)	<0.001	-0.256	<0.001	0.335	0.082
Lys	5.4 (0.15)	5.4 (0.16)	0.841	0.053	0.419	0.197	0.018
Met	3.43 (0.15)	3.33 (0.15)	<0.001	-0.133	0.043	0.639	0.023
N-Acetyl-phenylalanine	-3.65 (0.45)	-3.83 (0.45)	0.003	-0.101	0.126	0.562	0.700
Orn	4.22 (0.25)	4.17 (0.26)	0.208	-0.039	0.554	0.354	0.069
Phe	4.41 (0.12)	4.36 (0.13)	0.002	-0.201	0.002	0.873	0.072
Pro	5.5 (0.27)	5.38 (0.25)	<0.001	-0.023	0.732	0.062	0.290
Put	-1.49 (0.61)	-1.5 (0.71)	0.952	0.097	0.143	0.541	0.172
Sarco	0.6 (0.3)	0.44 (0.33)	<0.001	-0.131	0.047	0.150	0.947
Ser	4.5 (0.19)	4.49 (0.22)	0.860	-0.004	0.952	0.406	0.244
Serotonin	-1.13 (0.82)	-1.35 (1.12)	0.108	0.012	0.852	0.525	0.747
Tau	4.72 (0.28)	4.81 (0.23)	0.006	-0.110	0.097	0.418	0.951
Thr	4.88 (0.2)	4.81 (0.23)	0.025	-0.082	0.217	0.379	0.160
Trp	4.33 (0.17)	4.22 (0.19)	<0.001	-0.164	0.013	0.776	0.296
Typtamine	-3.86 (0.45)	-4.06 (0.49)	0.002	-0.044	0.508	0.535	0.315
Tyr	4.53 (0.22)	4.47 (0.22)	0.037	-0.112	0.088	0.030	0.231
Val	5.65 (0.18)	5.56 (0.18)	<0.001	-0.197	0.003	0.163	0.757

Supplementary Table 3. Mean ratios/interaction levels between CN and iPD groups. To assess the interrelationships between metabolites, all possible metabolite ratios and interactions were calculated for assessment between the CN and treated iPD groups.

Metabolite	N				Mean (SD)				unadjusted for coundounders p-values					Adjusted for confounders p-values		
	CN	naive-PD	iPD	AD	CN	naive-PD	iPD	AD	naive-PD vs iPD	naïve-PD vs CN	iPD vs CN	AD vs CN	iPD vs AD	iPD vs CN	AD vs CN	iPD vs AD
Homoserine*Orn	93	7	103	28	23.77 (11.96)	28.84 (15.45)	31.98 (11.89)	26.15 (11.49)	0.515	0.310	1.00E-06	0.262	3.17E-02	1.30E-05	0.239	1.74E-02
β-ala*Orn	93	7	103	28	297.95 (156.41)	336.65 (251.12)	398.66 (181.81)	259.8 (103.45)	0.324	0.845	7.00E-06	0.336	4.50E-05	1.50E-05	0.783	8.30E-05
Asp*Tyr	93	7	103	28	845.07 (479.13)	974.6 (531.85)	1278.56 (593.25)	772.85 (346.92)	0.295	0.770	1.00E-06	0.947	2.50E-05	1.90E-05	0.855	5.30E-05
Gln*Tyr	93	7	103	28	4001.77 (2008.08)	4756.41 (1511.22)	5695.77 (2774.62)	3026.21 (1297.06)	0.430	0.109	1.00E-06	0.013	0.00E+00	2.10E-05	0.038	6.00E-06
Asp*Orn	93	7	103	28	646.85 (407.25)	780.03 (542.86)	951.5 (464.85)	611.64 (277.79)	0.349	0.852	2.00E-06	0.626	5.10E-04	4.10E-05	0.799	1.80E-04
Asp/L-KYN	93	7	103	28	3.42 (1.97)	4.82 (3.49)	5.03 (2.33)	4.12 (2.28)	0.558	0.434	0.00E+00	0.091	4.58E-02	4.30E-05	0.151	2.22E-02
Gln*Orn	93	7	103	28	3036.22 (1674.92)	3564.5 (1613.55)	4207.2 (2027.61)	2423.13 (1117.07)	0.463	0.277	3.00E-06	0.123	6.00E-06	4.90E-05	0.194	2.70E-05
Homoserine*Asp	93	7	103	28	3.72 (2.8)	4.27 (2.24)	5.28 (2.56)	4.36 (2.89)	0.412	0.399	7.00E-06	0.137	8.83E-02	8.70E-05	0.239	1.10E-02
Homoserine*Gln	93	7	103	28	17.14 (11.2)	21.68 (5.6)	23.5 (12.31)	16.13 (8.18)	0.923	0.010	8.00E-06	0.973	2.37E-03	1.03E-04	0.971	1.08E-03
Asp*β-ala	93	7	103	28	45.39 (30.63)	57.03 (43.98)	66.15 (37.25)	40.47 (19.1)	0.406	0.848	1.20E-05	0.877	3.12E-04	1.15E-04	0.793	1.45E-04
Homoserine*β-ala	93	7	103	28	1.72 (1.09)	1.99 (1.01)	2.24 (1.09)	1.78 (0.85)	0.592	0.296	7.00E-05	0.520	4.29E-02	1.56E-04	0.300	9.20E-03
Gln*β-ala	93	7	103	28	217.91 (145.68)	253.6 (126.92)	292.63 (173.49)	159.33 (73.78)	0.590	0.324	4.50E-05	0.062	2.00E-06	1.67E-04	0.234	3.40E-05
Arg/Asp	93	7	103	28	12.39 (15.1)	10.22 (7.07)	7.19 (4.72)	10.28 (5.39)	0.321	0.657	2.00E-06	0.734	1.27E-03	1.82E-04	0.884	9.75E-04
Asp/Cys	93	7	103	28	0.06 (0.03)	0.07 (0.03)	0.08 (0.04)	0.05 (0.02)	0.361	0.589	1.00E-06	0.979	3.40E-05	2.59E-04	0.930	1.39E-04
Asp/2-Ambut	93	7	103	28	0.48 (0.28)	0.48 (0.3)	0.64 (0.32)	0.56 (0.29)	0.228	0.907	6.30E-05	0.070	2.32E-01	3.16E-04	0.182	1.16E-01
Asp*Gln	93	7	103	28	503 (480.26)	619.4 (398.59)	723.85 (477.04)	405.28 (280.77)	0.535	0.525	3.10E-05	0.684	3.46E-04	3.74E-04	0.611	1.21E-04
Asp/Lys	93	7	103	28	0.04 (0.02)	0.05 (0.03)	0.06 (0.02)	0.05 (0.02)	0.447	0.780	3.40E-05	0.134	4.70E-02	5.19E-04	0.361	2.27E-02
Asp*Thr	93	7	103	28	1258.72 (763.75)	1328.03 (714.45)	1725.4 (802.02)	1214.27 (599.01)	0.305	0.922	3.80E-05	0.605	2.78E-03	5.49E-04	0.714	1.46E-03
Orn*3-HK	93	7	103	28	2.28 (1.29)	1.92 (1.18)	3.46 (2.14)	1.58 (0.69)	0.066	0.480	3.90E-05	0.005	0.00E+00	6.31E-04	0.017	4.29E-04
Gln/Asp	93	7	103	28	111.76 (150.06)	103.34 (90.43)	70.03 (51.09)	85.93 (43.33)	0.442	0.787	8.50E-05	0.449	1.21E-02	7.27E-04	0.781	1.83E-03
Gln*Thr	93	7	103	28	5818.59 (2990.61)	6358.46 (1537.65)	7709.95 (3964.18)	4873.56 (2521.66)	0.351	0.138	4.50E-05	0.113	7.70E-05	7.56E-04	0.176	2.68E-04
Orn*hydroxy.antranilic.acid	93	7	103	28	2.27 (0.99)	2.18 (0.99)	3.16 (1.59)	1.71 (0.73)	0.087	0.968	8.70E-05	0.010	1.00E-06	8.94E-04	0.061	1.33E-04
Asp*3-HK	93	7	103	28	0.37 (0.37)	0.32 (0.21)	0.57 (0.37)	0.24 (0.12)	0.155	0.712	3.00E-05	0.158	1.00E-06	9.15E-04	0.174	3.70E-04
Tyr*3-HK	93	7	103	28	3.12 (1.81)	2.48 (1.15)	4.71 (3.07)	2.03 (0.89)	0.028	0.404	5.50E-05	0.001	0.00E+00	1.03E-03	0.005	2.08E-04
Asp*Pro	93	7	103	28	2244.92 (1282.91)	2912.11 (2002.8)	3027.73 (1527.01)	2406.65 (1020.46)	0.643	0.491	6.50E-05	0.141	5.33E-02	1.03E-03	0.225	9.96E-03
Tyr*N-Acetyl-phenylalanine	93	7	103	28	2.27 (1.24)	1.92 (0.43)	2.93 (1.29)	1.55 (0.61)	0.005	0.646	6.90E-05	0.002	0.00E+00	1.08E-03	0.021	1.20E-05
Asp/Put	93	7	103	28	68.22 (188.83)	56.24 (39.63)	100.4 (148.22)	43.58 (30.65)	0.339	0.738	6.90E-05	0.931	1.43E-03	1.29E-03	0.953	3.25E-03
Asp*hydroxy.anthranilic.acid	93	7	103	28	0.35 (0.24)	0.38 (0.29)	0.52 (0.34)	0.26 (0.12)	0.258	0.942	4.90E-05	0.197	2.00E-06	1.29E-03	0.358	1.10E-04
Gln*3-HK	93	7	103	28	1.82 (1.94)	1.48 (0.74)	2.41 (1.41)	1.04 (0.7)	0.113	0.966	8.60E-05	0.005	0.00E+00	1.45E-03	0.010	4.30E-05
Orn/Cad	93	7	103	28	423.33 (376.29)	588.37 (285.68)	549.44 (297.14)	319.84 (107.77)	0.584	0.052	3.90E-05	0.113	1.00E-06	1.63E-03	0.297	3.41E-04
Asp*N-Acetyl-phenylalanine	93	7	103	28	0.26 (0.21)	0.24 (0.15)	0.37 (0.21)	0.2 (0.12)	0.175	0.911	5.30E-05	0.325	8.20E-05	1.76E-03	0.394	1.61E-04
L-KYN/3-HK	93	7	103	28	104.79 (62.17)	98.49 (35.21)	76.58 (47.88)	115.67 (44.81)	0.034	0.978	4.00E-06	0.135	3.00E-06	1.78E-03	0.157	6.85E-03
Asp/Cad	93	7	103	28	69.69 (84.73)	97.8 (71.97)	90.49 (58.89)	50.45 (24.12)	0.992	0.196	6.60E-05	0.769	3.10E-05	2.37E-03	0.745	3.51E-04
Asp/Citrulline	93	7	103	28	0.29 (0.17)	0.35 (0.21)	0.4 (0.19)	0.28 (0.14)	0.479	0.672	7.80E-05	0.863	4.67E-03	3.99E-03	0.915	6.26E-03

Supplementary Table 4. Mean ratios/interaction levels between AD and iPD. Same assessment as in Supplementary Table 2.

Metabolite	N				Mean (SD)				unadjusted for coundounders p-values					Adjusted for confounders p-values		
	CN	naïve-PD	iPD	AD	CN	naïve-PD	iPD	AD	naïve-PD vs iPD	naïve-PD vs CN	iPD vs CN	AD vs CN	iPD vs AD	iPD vs CN	AD vs CN	iPD vs AD
Gln*Tyr	93	7	103	28	4001.77 (2008.08)	4756.41 (1511.22)	5695.77 (2774.62)	3026.21 (1297.06)	0.429841	0.109172	1.00E-06	0.013301	0	2.10E-05	0.038469	6.00E-06
Cad/N-Acetyl-phenylalanine	93	7	103	28	9.67 (6.2)	7.01 (5.54)	7.22 (5.23)	12.1 (5.69)	0.953762	0.283706	0.004548	0.00478	0	0.078795	0.057181	0.000458
Phe*3-HK	93	7	103	28	2.9 (1.67)	2.22 (0.96)	3.82 (2.19)	1.95 (0.8)	0.040634	0.297522	0.003536	0.001294	0	0.031236	0.008569	0.001205
Asn*3-HK	93	7	103	28	1.5 (0.87)	1.17 (0.58)	1.91 (1.06)	0.99 (0.4)	0.05412	0.319379	0.006118	0.000479	0	0.040923	0.006113	0.000924
Gly/3-HK	93	7	103	28	25848.96 (14397.63)	29390.23 (12533.15)	21150.56 (13713.42)	31747.96 (12096.33)	0.053147	0.344501	0.003957	0.00322	0	0.025656	0.008913	0.00334
Trp*3-HK	93	7	103	28	2.69 (1.54)	2.15 (0.92)	3.38 (1.92)	1.69 (0.79)	0.083877	0.391153	0.017027	0.000162	0	0.129541	0.001766	0.001963
Tyr*3-HK	93	7	103	28	3.12 (1.81)	2.48 (1.15)	4.71 (3.07)	2.03 (0.89)	0.027519	0.403836	5.50E-05	0.000946	0	0.001027	0.004979	0.000208
Val*3-HK	93	7	103	28	10.08 (6.25)	7.99 (3.4)	12.85 (7.47)	6.47 (3.14)	0.065714	0.421249	0.008483	0.000597	0	0.070434	0.0041	0.001032
Leu*3-HK	93	7	103	28	5.72 (3.64)	4.53 (1.8)	7.52 (4.55)	3.64 (1.79)	0.052989	0.442073	0.004011	0.000537	0	0.055787	0.005586	0.000906
β-ala*3-HK	93	7	103	28	0.17 (0.13)	0.13 (0.07)	0.23 (0.15)	0.11 (0.06)	0.06062	0.472436	0.000287	0.006723	0	0.001676	0.037271	0.000164
Orn*3-HK	93	7	103	28	2.28 (1.29)	1.92 (1.18)	3.46 (2.14)	1.58 (0.69)	0.065596	0.480323	3.90E-05	0.004936	0	0.000631	0.017262	0.000429
Cys/3-HK	93	7	103	28	6398.49 (3682.97)	6700.56 (2925.6)	4887.63 (3341.8)	8426.02 (2839.71)	0.027059	0.498573	0.000114	0.000139	0	0.015177	0.003464	0.000729
Tyr*N-Acetyl-phenylalanine	93	7	103	28	2.27 (1.24)	1.92 (0.43)	2.93 (1.29)	1.55 (0.61)	0.005488	0.645799	6.90E-05	0.00193	0	0.001083	0.021083	1.20E-05
Cys/3-AA	93	7	103	28	6186.13 (5265.12)	5311.31 (2034.6)	4643.32 (2416.73)	7930.6 (3203.45)	0.320297	0.685893	0.000427	0.000782	0	0.044868	0.018668	0.000118
Cys/N-Acetyl-phenylalanine	93	7	103	28	8623.12 (4711.73)	7829.53 (2482.68)	6474.26 (3256.57)	11065.7 (4615.72)	0.107346	0.949113	0.000313	0.00107	0	0.046458	0.018842	0.000139
Gln*3-HK	93	7	103	28	1.82 (1.94)	1.48 (0.74)	2.41 (1.41)	1.04 (0.7)	0.112678	0.966084	8.60E-05	0.004892	0	0.001452	0.010409	4.30E-05
Orn/Cad	93	7	103	28	423.33 (376.29)	588.37 (285.68)	549.44 (297.14)	319.84 (107.77)	0.583538	0.051845	3.90E-05	0.112686	1.00E-06	0.001633	0.297439	0.000341
3-HK*N-Acetyl-phenylalanine	93	7	103	28	0.001069 (0.001035)	0.000583 (0.000245)	0.001391 (0.000991)	0.000548 (0.000365)	0.006541	0.141354	0.004538	0.002334	1.00E-06	0.035667	0.011182	0.000699
Thr*3-HK	93	7	103	28	4.5 (2.7)	3.36 (1.62)	6.31 (3.95)	3.18 (1.67)	0.023122	0.24121	0.000692	0.004898	1.00E-06	0.008525	0.016323	0.001826
Met*3-HK	93	7	103	28	1.08 (0.62)	0.82 (0.31)	1.38 (0.8)	0.72 (0.32)	0.039126	0.272283	0.008666	0.001225	1.00E-06	0.061353	0.007175	0.00166
Arg/3-HK	93	7	103	28	2931.38 (1651.62)	3064.42 (992.07)	2288.53 (1570.57)	3913.32 (2138.55)	0.010803	0.317445	0.000544	0.00438	1.00E-06	0.01425	0.013294	0.002974
3-HK*3-AA	93	7	103	28	0.00143 (0.00119)	0.000956 (0.000668)	0.00201 (0.00161)	0.000762 (0.000515)	0.037884	0.336242	0.005973	0.001668	1.00E-06	0.037652	0.015578	0.000491
Cad/3-HK	93	7	103	28	7.26 (5.4)	5.08 (2.25)	5.48 (4.98)	10.3 (8.43)	0.392329	0.339559	0.001312	0.005529	1.00E-06	0.026388	0.019806	0.000806
Ile*3-HK	93	7	103	28	2.92 (1.91)	2.22 (0.85)	3.83 (2.31)	1.94 (0.99)	0.042327	0.367257	0.003828	0.003237	1.00E-06	0.029495	0.015047	0.001511
3-HK*Tryptamine	93	7	103	28	0.000801 (0.000651)	0.000563 (0.000281)	0.00103 (0.000743)	0.000423 (0.000244)	0.055771	0.390078	0.019616	0.000359	1.00E-06	0.083061	0.002841	0.000792
Gln*3-AA	93	7	103	28	1.68 (1.19)	1.86 (1.12)	2.38 (1.6)	1.1 (0.66)	0.437945	0.413988	0.000308	0.004406	1.00E-06	0.003654	0.01639	4.80E-05
Citrulline/3-HK	93	7	103	28	1246.18 (727.42)	1348.03 (617.07)	997.43 (622.64)	1669.81 (820.34)	0.074145	0.469849	0.003778	0.002422	1.00E-06	0.084543	0.01113	0.004335
Put/3-HK	93	7	103	28	10.58 (7.67)	9.99 (5.73)	8.4 (10.06)	12.7 (7.6)	0.033521	0.583456	0.001762	0.019651	1.00E-06	0.013834	0.07132	0.002813
Aspartic acid*3-HK	93	7	103	28	0.37 (0.37)	0.32 (0.21)	0.57 (0.37)	0.24 (0.12)	0.154509	0.712281	3.00E-05	0.157868	1.00E-06	0.000915	0.174031	0.00037
Arg/N-Acetyl-phenylalanine	93	7	103	28	3926.17 (2159.37)	3715.33 (1335.45)	3003.72 (1547.55)	4906.1 (2164.19)	0.122444	0.877465	0.001112	0.006758	1.00E-06	0.037353	0.050129	0.000546
Citrulline/N-Acetyl-phenylalanine	93	7	103	28	1671.7 (912.62)	1564.02 (558.53)	1328.1 (615.28)	2079.3 (826.09)	0.225429	0.907522	0.01352	0.002205	1.00E-06	0.280638	0.03978	0.000834
Tyr*3-AA	93	7	103	28	3.18 (1.52)	3.05 (1.46)	4.28 (2.26)	2.23 (1.03)	0.116261	0.931885	0.000274	0.003653	1.00E-06	0.002551	0.027848	5.10E-05
Orn*3-AA	93	7	103	28	2.27 (0.99)	2.18 (0.99)	3.16 (1.59)	1.71 (0.73)	0.087234	0.96763	8.70E-05	0.010214	1.00E-06	0.000894	0.061088	0.000133
Gln/Cad	93	7	103	28	303.64 (252.81)	485.92 (228.02)	394.57 (227.23)	207.4 (131.79)	0.274427	0.022835	0.000101	0.031859	2.00E-06	0.004261	0.090504	6.20E-05
Gln*Tryptamine	93	7	103	28	0.99 (0.73)	1.06 (0.27)	1.26 (0.81)	0.62 (0.36)	0.871071	0.052914	0.004258	0.001814	2.00E-06	0.018255	0.008659	6.40E-05
Gln*β-ala	93	7	103	28	217.91 (145.68)	253.6 (126.92)	292.63 (173.49)	159.33 (73.78)	0.589508	0.324185	4.50E-05	0.062006	2.00E-06	0.000167	0.234291	3.40E-05
Orn*N-Acetyl-phenylalanine	93	7	103	28	1.66 (0.9)	1.43 (0.64)	2.21 (1.03)	1.22 (0.48)	0.031697	0.652049	8.90E-05	0.020873	2.00E-06	0.001138	0.080293	0.000133
Aspartic acid*3-AA	93	7	103	28	0.35 (0.24)	0.38 (0.29)	0.52 (0.34)	0.26 (0.12)	0.257562	0.941777	4.90E-05	0.19695	2.00E-06	0.001286	0.35838	0.00011
Pro/3-HK	93	7	103	28	8238.42 (5364.92)	11311.22 (4990.47)	7613.87 (5203.18)	10877.88 (4732.36)	0.024345	0.076968	0.101548	3.05E-04	3.00E-06	0.158969	0.001566	0.00847
N-Acetyl-phenylalanine*3-AA	93	7	103	28	0.001 (0.000754)	0.000759 (0.00038)	0.00128 (0.000878)	0.000578 (0.000381)	0.097368	0.591552	0.010025	0.002194	3.00E-06	0.067929	0.019164	0.000294
Leu*N-Acetyl-phenylalanine	93	7	103	28	4.14 (2.35)	3.7 (1.3)	4.86 (2.31)	2.79 (1.21)	0.183035	0.914658	0.01471	0.001912	3.00E-06	0.176846	0.026441	0.000448
L-KYN/3-HK	93	7	103	28	104.79 (62.17)	98.49 (35.21)	76.58 (47.88)	115.67 (44.81)	0.034168	0.977929	4.00E-06	0.134862	3.00E-06	0.001779	0.156997	0.006853
Lys/3-HK	93	7	103	28	8300.6 (4861.08)	9354.34 (3955.75)	6796.95 (4315.02)	9405.52 (3190.14)	0.04445	0.334256	0.003307	0.019449	4.00E-06	0.02383	0.042697	0.016842
Gln*N-Acetyl-phenylalanine	93	7	103	28	1.24 (0.97)	1.16 (0.44)	1.63 (0.92)	0.8 (0.48)	0.196697	0.527486	0.000326	0.010359	4.00E-06	0.004118	0.031017	4.60E-05
Trp*N-Acetyl-phenylalanine	93	7	103	28	1.97 (1.08)	1.67 (0.34)	2.19 (0.97)	1.29 (0.5)	0.084184	0.669714	0.068393	0.000579	4.00E-06	0.417407	0.009936	0.000931
Sarco*3-HK	93	7	103	28	0.06 (0.04)	0.06 (0.04)	0.08 (0.06)	0.04 (0.02)	0.196575	0.713436	0.014357	0.003344	5.00E-06	0.138804	0.018732	0.008118
Cad/Tyr	93	7	103	28	0.00238 (0.00107)	0.00149 (0.000808)	0.00175 (0.000936)	0.0029 (0.00151)	0.492203	0.051878	0.000104	0.048372	6.00E-06	0.004528	0.133011	0.000215
Gln*Orn	93	7	103	28	3036.22 (1674.92)	3564.5 (1613.55)	4207.2 (2027.61)	2423.13 (1117.07)	0.463051	0.276786	3.00E-06	0.123387	6.00E-06	4.90E-05	0.194467	2.70E-05
His*3-HK	93	7	103	28	2.54 (1.42)	2.01 (0.72)	3.21 (1.85)	1.79 (0.8)	0.050759	0.40129	0.012175	0.005963	6.00E-06	0.074948	0.027765	0.004739
Gln*Leu	93	7	103	28	7404.65 (4171.7)	9156.52 (3621.55)	9235.58 (4493.86)	5500.41 (2688.68)	0.871337	0.140731	0.000675	0.015704	7.00E-06	0.011445	0.06131	0.000102
Gly/3-HK	93	7	103	28	9881.47 (5522.65)	11454.21 (5059.55)	8971.94 (5991.74)	13150.68 (5491.54)	0.154258	0.377303	0.110062	0.000654	7.00E-06	0.396266	0.004143	0.018546
Thr*3-AA	93	7	103	28	4.55 (2.02)	3.93 (0.81)	5.7 (2.71)	3.4 (1.47)	0.010941	0.601224	0.002661	0.009227	7.00E-06	0.020891	0.071985	0.000678
Gln/Put	93	7	103	28	301.94 (732.56)	257.28 (115.06)	430.48 (565.57)	160.08 (86.8)	0.47898	0.217347	0.00037	0.110822	8.00E-06	0.003408	0.246435	0.000483
Ser*3-HK	93	7	103	28	3.18 (1.78)	2.39 (1.09)	4.14 (2.24)	2.35 (1.05)	0.045942	0.263255	0.004096	0.014253	8.00E-06	0.020226	0.03448	0.005758
Ala*3-HK	93	7	103	28	15.12 (9.49)	12.54 (6.88)	19.22 (10.64)	10.68 (5.1)	0.126161	0.504828	0.003343	0.011016	8.00E-06	0.017963	0.019634	0.000746
Put/3-AA	93	7	103	28	9.73 (8.04)	7.82 (4.04)	7.63 (9.78)	12.05 (9)	0.183037	0.807811	0.002224	0.028937	8.00E-06	0.018615	0.136241	0.001359
Val*N-Acetyl-phenylalanine	93	7	103	28	7.28 (4)	6.38 (1.8)	8.37 (3.85)	4.95 (2.12)	0.149701	0.839714	0.032202	0.001921	8.00E-06	0.229127	0.019485	0.000571
Cys*Cad	93	7	103	28	36.02 (19.34)	21.59 (13.45)	26.8 (13.7)	41.96 (20.68)	0.32083	0.054959	0.000705	0.049001	9.00E-06	0.092863	0.157606	0.001507
β-ala*N-Acetyl-phenylalanine	93	7	103	28	0.12 (0.09)	0.11 (0.06)	0.15 (0.08)	0.08 (0.04)	0.129448	0.777164	0.001083	0.023818	9.00E-06	0.003528	0.147281	4.80E-05
Phe*N-Acetyl-phenylalanine	93	7	103	28	2.12 (1.18)	1.76 (0.52)	2.46 (1.09)	1.51 (0.58)	0.05781	0.528188	0.013562	0.005886	1.00E-05	0.101376	0.045162	0.000429
Tau*3-HK	93	7	103	28	4.05 (2.57)	3.43 (1.51)	5.64 (3.33)	3.05 (1.12)	0.110302	0.681413	0.000601	0.093492	1.00E-05	0.006079	0.110813	0.001671
His/3-HK	93	7	103	28	2540.01 (1491.91)	3264.3 (1559.73)	2157.99 (1472.52)	3094.78 (1251.74)	0.036043	0.168984	0.013288	0.008256	1.20E-05	0.035078	0.011359	0.006816
Thr*N-Acetyl-phenylalanine	93	7	103	28	3.26 (1.7)	2.59 (0.48)	4.08 (1.92)	2.42 (1)	0.001279	0.216968	0.002702	0.014635	1.20E-05	0.027611	0.074218	0.000961
Lys*3-HK	93	7	103	28	8.27 (4.86)	5.94 (2.25)	10.02 (5.69)	5.62 (2.55)	0.033567	0.167842	0.035394	0.002277	1.30E-05	0.092889	0.005932	0.001808
Asn*3-AA	93	7	103	28	1.51 (0.62)	1.36 (0.24)	1.76 (0.79)	1.08 (0.44)	0.060623	0.90475	0.03164	0.002676	1.30E-05	0.133535	0.036305	0.000411
Leu*3-AA	93	7	103	28	5.78 (2.93)	5.79 (2.83)	7.09 (3.94)	3.99 (1.96)	0.462332	0.796057	0.023805	0.002536	1.50E-05	0.185994	0.026081	0.00085
Ser/3-HK	93	7	103	28	3248.68 (1889.59)	3615.51 (1518.01)	2801.41 (1673.81)	4098.81 (1714.24)	0.065587	0.301325	0.040699	0.004076	1.70E-05	0.133817	0.010791	0.00787
Gly*3-HK	93	7	103	28	25.32 (13.7)	18.62 (7.33)	31.2 (17.53)	18.38 (7.88)	0.035091	0.178775	0.024594	0.006014	1.80E-05	0.078563	0.021655	0.007216
Ala/3-HK	93	7	103	28	14898.76 (9211.42)	17544.18 (5029.36)	13457.52 (9520.16)	17748.22 (5994.8)	0.014448	0.095577	0.046236	0.004388	1.90E-05	0.140332	0.016582	0.042534
2-Ambut*3-HK	93	7	103	28	0.77 (0.47)	0.69 (0.46)	0.93 (0.58)	0.5 (0.24)	0.269419	0.610942	0.059571	0.001282	2.20E-05	0.243591	0.002118	0.001449
β-ala*3-AA	93	7	103	28	0.16 (0.09)	0.16 (0.08)	0.22 (0.12)	0.12 (0.06)	0.189766	0.998428	0.001046	0.018999	2.20E-05	0.003219	0.096979	0.000139
Phe*3-AA	93	7	103	28	2.93 (1.33)	2.78 (1.36)	3.57 (1.86)	2.14 (0.98)	0.270832	0.912099	0.020277	0.005257	2.30E-05	0.109671	0.042357	0.000763
N-Acetyl-phenylalanine*Tryptamine	93	7	103	28	0.000549 (0.000357)	0.000472 (0.000228)	0.000683 (0.000513)	0.000333 (0.000202)	0.255415	0.791392	0.039253	0.001534	2.40E-05	0.160935	0.005783	0.000398
Aspartic acid*Tyr	93	7	103	28	845.07 (479.13)	974.6 (531.85)	1278.56 (593.25)	772.85 (346.92)	0.295165	0.770235	1.00E-06	0.946736	2.50E-05	1.90E-05	0.85482	5.30E-05
Gly*3-HK	93	7	103	28	9.81 (5.73)	7.24 (3.23)	13.62 (8.75)	7.55 (3.16)	0.023418	0.234005	0.001801	0.061154	2.70E-05	0.005688	0.083401	0.004044
Gln*Val	93	7	103	28	13050.45 (7106.07)	15783.17 (5522.65)	15977.96 (7789.44)	9827.11 (4812.04)	0.769513	0.107234	0.002156	0.019098	2.80E-05	0.017195	0.047302	0.000146
Aspartic acid/Cad	93	7	103	28	69.69 (84.73)	97.8 (71.97)	90.49 (58.89)	50.45 (24.12)	0.992424	0.195539	6.60E-05	0.768588	3.10E-05	0.002372	0.74455	0.000351
Arg/3-AA	93	7	103	28	2750.64 (2027.48)	2525.16 (1080.16)	2208.56 (1326.77)	3677.37 (2054.34)	0.325034	0.839025	0.001654	0.012434	3.30E-05	0.038193	0.04057	0.001294
Aspartic acid/Cys	93	7	103	28	0.06 (0.03)	0.07 (0.03)	0.08 (0.04)	0.05 (0.02)	0.361129	0.58866	1.00E-06	0.97942	3.40E-05	0.000259	0.929784	0.000139
GABA/3-HK	93	7	103	28	11.35 (8.85)	12.98 (5.59)	9.47 (7.39)	15.88 (9.26)	0.020993	0.137992	0.051481	0.004536	3.60E-05	0.309533	0.02193	0.038071
Trp*3-AA	93	7	103	28	2.77 (1.35)	2.66 (1.22)	3.15 (1.59)	1.87 (0.86)	0.496499	0.896058	0.097389	0.001422	3.60E-05	0.422417	0.012592	0.001537
Val*3-AA	93	7	103	28	10.16 (4.92)	10.11 (4.96)	12.26 (6.78)	7.1 (3.41)	0.495571	0.828068	0.050429	0.00263	3.70E-05	0.245297	0.019078	0.00125
β-ala/Cad	93	7	103	28	30.21 (32.66)	44.57 (29.08)	37.28 (20.28)	22.31 (10.77)	0.626654	0.133771	0.000198	0.10445	4.00E-05	0.003826	0.34022	0.000128
Met*N-Acetyl-phenylalanine	93	7	103	28	0.79 (0.43)	0.65 (0.16)	0.9 (0.41)	0.56 (0.23)	0.044614	0.480345	0.037792	0.00548	4.50E-05	0.208362	0.037469	0.001044
β-ala*Orn	93	7	103	28	297.95 (156.41)	336.65 (251.12)	398.66 (181.81)	259.8 (103.45)	0.324087	0.845322	7.00E-06	0.335546	4.50E-05	1.50E-05	0.782911	8.30E-05
4-OH-Pro/3-HK	93	7	103	28	415.34 (307.73)	562.19 (229.71)	357.87 (226.98)	528.65 (252.25)	0.039745	0.099694	0.159459	0.002561	4.60E-05	0.27719	0.004513	0.021063
Ile*3-AA	93	7	103	28	2.93 (1.44)	2.77 (1.15)	3.62 (2.02)	2.11 (1.04)	0.290957	0.919057	0.023551	0.008098	4.70E-05	0.103393	0.058277	0.001218
Ile*N-Acetyl-phenylalanine	93	7	103	28	2.1 (1.21)	1.78 (0.5)	2.48 (1.2)	1.52 (0.81)	0.075219	0.715886	0.013073	0.012133	5.10E-05	0.0901	0.064255	0.000912
Put/Tyr	93	7	103	28	0.0034 (0.00186)	0.00263 (0.000857)	0.00255 (0.00194)	0.00388 (0.00243)	0.09805	0.404442	0.000316	0.192363	5.30E-05	0.002587	0.384697	0.00169
Gln*Ile	93	7	103	28	3752.21 (2118.63)	4445.18 (1615.1)	4697.72 (2273.05)	2936.21 (1469.98)	0.952654	0.173054	0.000595	0.054998	5.50E-05	0.004456	0.129784	0.000204
Met*3-AA	93	7	103	28	1.1 (0.5)	1 (0.3)	1.28 (0.63)	0.79 (0.36)	0.200855	0.910281	0.049495	0.004675	5.80E-05	0.209935	0.040596	0.001127
Cad/3-AA	93	7	103	28	7.14 (7.59)	4.88 (4.18)	5.1 (5.11)	9.98 (7.93)	0.784315	0.253878	0.003778	0.021202	5.90E-05	0.064686	0.068951	0.000332
Tyr*Tryptamine	93	7	103	28	1.83 (0.88)	1.91 (0.84)	2.35 (1.36)	1.35 (0.73)	0.450734	0.641202	0.005873	0.00645	6.10E-05	0.017291	0.009161	0.000262
Cad/Leu	93	7	103	28	0.0013 (0.000571)	0.000838 (0.000558)	0.00108 (0.000573)	0.00163 (0.000863)	0.26448	0.07632	0.009118	0.025489	6.20E-05	0.222823	0.134688	0.002252
Ala*3-AA	93	7	103	28	15.05 (6.94)	14.32 (5.14)	18.24 (9.72)	11.38 (5.01)	0.269676	0.901188	0.018993	0.013254	6.40E-05	0.067935	0.06525	0.000573
Citrulline/3-AA	93	7	103	28	1212.4 (1024.35)	1079.73 (462.3)	971.76 (523.91)	1573.69 (821.84)	0.547544	0.838396	0.024221	0.009028	6.90E-05	0.29929	0.054399	0.003907
Ala*N-Acetyl-phenylalanine	93	7	103	28	11.02 (6.39)	9.34 (2.9)	12.74 (5.96)	8.15 (3.77)	0.081225	0.714903	0.017487	0.02154	7.50E-05	0.070426	0.082104	0.000335
Sarco*N-Acetyl-phenylalanine	93	7	103	28	0.05 (0.03)	0.04 (0.02)	0.05 (0.03)	0.03 (0.02)	0.468696	0.83369	0.061018	0.00978	7.60E-05	0.400022	0.08241	0.009418
Gln*Thr	93	7	103	28	5818.59 (2990.61)	6358.46 (1537.65)	7709.95 (3964.18)	4873.56 (2521.66)	0.351404	0.138419	4.50E-05	0.113124	7.70E-05	0.000756	0.175657	0.000268
Met/3-HK	93	7	103	28	1079.31 (609.97)	1286.32 (536.28)	923.11 (581.93)	1225.98 (460.06)	0.045689	0.232402	0.015628	0.034418	7.80E-05	0.037489	0.032448	0.019284
Aspartic acid*N-Acetyl-phenylalanine	93	7	103	28	0.26 (0.21)	0.24 (0.15)	0.37 (0.21)	0.2 (0.12)	0.175386	0.910972	5.30E-05	0.324849	8.20E-05	0.001756	0.394334	0.000161
Phe/3-HK	93	7	103	28	2870.14 (1670.86)	3400.74 (1382.76)	2505.74 (1508.61)	3369.97 (1263.59)	0.048549	0.215443	0.030136	0.017662	8.40E-05	0.068892	0.024016	0.021839
Aspartic acid*Tryptamine	93	7	103	28	0.21 (0.16)	0.21 (0.13)	0.29 (0.2)	0.16 (0.1)	0.353394	0.749135	0.000882	0.178637	8.70E-05	0.007888	0.265859	3.00E-04
